# Quantifying the strength of firearms comparisons based on error rate studies

**DOI:** 10.1111/1556-4029.15646

**Published:** 2024-10-30

**Authors:** Nada Aggadi, Kimberley Zeller, Tom Busey

**Affiliations:** ^1^ Department of Psychological and Brain Sciences Indiana University‐Bloomington Bloomington Indiana USA; ^2^ Houston Forensic Science Center Houston Texas USA

**Keywords:** calibration, error rates, firearm evidence, likelihood ratios, ordered probit model, strength of evidence

## Abstract

Forensic firearms and tool mark examiners compare bullets and cartridge cases to assess whether they originate from the same source or different sources. To communicate their observations, they rely on predefined conclusion scales ranging from Identification to Elimination. However, these terms have not been calibrated against the actual strength of the evidence except indirectly through error rate studies. The present research reanalyzes the findings of firearms and cartridge case comparisons from error rate studies to generate a quantitative measure of the strength of the evidence for each comparison. We use an ordered probit model to summarize the distribution of responses of examiners and aggregate the data for all comparisons to produce a set of likelihood ratios. The likelihood ratios can be as low as less than 10, which does not seem to justify the current articulation scale that may imply a strength of evidence of 10,000 or greater. This suggests that examiners are using language that overstates the strength of the evidence by several orders of magnitude.

**Highlights**

- An ordered probit model was used to generate likelihood ratios for firearms evidence.
- The current verbal conclusion scale was demonstrated to overstate the strength of evidence.
- Propose the use of likelihood ratios to quantify the strength of forensic evidence.
- Suggests that forensic examiners communicate their interpretation rather than make decisions.

## 1 INTRODUCTION

Firearms and toolmark examiners typically compare fired evidence such as bullets and cartridge cases found at crime scenes to a set of known test fires from a submitted firearm. Examiners have traditionally communicated their results using scales such as the Association of Firearm and Toolmark Examiners (AFTE) Range of Conclusions. This scale relies on the AFTE Glossary, which uses four terms: Identification, Inconclusive (subdivided into three subcategories), Elimination, and Unsuitable. The AFTE Glossary defines the terms as follows: Identification corresponds to an “agreement of a combination of individual characteristics and all discernable class characteristics where the extent of agreement exceeds that which can occur in the comparison of toolmarks made by different tools and is consistent with the agreement demonstrated by toolmarks known to have been produced by the same tool.” Elimination corresponds to a “significant disagreement of discernable class characteristics and/or individual characteristics” [1].

Although this model has historically been used in American forensic practice, many researchers have discussed the shortcomings of current scales used in forensic analysis [2], describing them as logically incorrect [2, 3]. To ask examiners to translate their observation to “Elimination, “Inconclusive,” or “Identification” is problematic and oversimplifies the strength of evidence [4] leaving space for individual differences in how those conclusions are reached and how they are interpreted by the public. There are five major problems with current practices. First, the verbal scale used in forensic firearms and tool mark examiners is a decision and therefore must respect the prior probability of a mated pair. In the extreme, if a police agency is under‐resourced and rarely submits a mated firearm, an examiner should rarely say Identification regardless of the similarity observed between samples. Second, when making a decision, experts have to take into account the consequences of making both erroneous identifications and erroneous eliminations (often term *utilities* in the field of decision‐making). Because examiners rarely have access to the full details of a case, it is difficult to know how an error might lead to a miscarriage of justice. Third, the way that laypersons interpret the articulation terms may not reflect the actual strength of evidence and could lead to judges and juries incorrectly interpreting it. For example, in fingerprint comparisons, 71% of laypersons believe that Identification means the exclusion of all others [5], which is not how many forensic examiners typically interpret the term. Fourth, the concept of “greater than closest nonmatch” is contradicted by a nonzero error rate in black box studies [6]. Finally, the current articulation language has not been calibrated against the actual strength of the evidence except indirectly through error rate studies [7, 8, 9]. We return to this last point in the Discussion, but we will demonstrate that current terms may overstate the strength of the evidence by up to five orders of magnitude.

A solution to the problems of current practice would be to allow examiners to provide a quantitative value describing the strength of evidence [10, 11, 12]. The approach described in the present work creates such a quantitative measure in the form of a likelihood ratio that converts words to numbers to create a scale that directly expresses the strength of the evidence. The current approach applies to data collected in error rate studies, and in the Discussion section we briefly describe extensions that could apply this method to casework.

The likelihood ratio is a ratio of the probability of an observation under two competing hypotheses or propositions [13]. The likelihood ratio is an important component in the theory of Bayesian belief updating as it allows new information to be combined with existing information [14]. Consider an example to illustrate Bayesian updating. Under this approach, experts estimate the likelihood of the observation given two alternative propositions: that the fired evidence was created by the same firearm (the same source proposition) and the fired evidence was created by different firearms (the different sources proposition). The ratio of these two probability densities is the likelihood ratio, and it is a measure of *evidential strength*. Importantly, the application of the likelihood ratio depends on the circumstances of the case. Suppose you have a likelihood ratio of 10,000 and a long list of 100,000 guns, each of which is equally likely to have been involved in the shooting (as might be the case if a search is run through the National Integrated Ballistic Information Network). Given no other information, every possible gun has a prior odds of 1/100,000. Multiplying the likelihood ratio by the prior odds would result in a posterior odds ratio of 1/10, which would not provide high posterior odds for any one gun as the source of the evidence because the prior odds can overwhelm the likelihood ratio. This situation exists even with very high likelihood ratios when very low prior odds could overwhelm even a high likelihood ratio to produce low posterior odds [15]. Now, let us suppose that we have 4 guns that could have possibly fired the evidence. Lacking other information, in this situation each gun would have a prior of ¼. Multiplying the likelihood ratio by the prior odds would result in a posterior odds ratio of 2500, which provides strong support for the proposition of which gun fired the bullet. This example illustrates how the priors can affect the interpretation of the observations and illustrates a fundamental problem with examiners making posterior conclusions without knowledge of the prior probability of a mated pair (the prior odds). In addition, examiners' role should not include estimating the prior probability of mated pairs, as this could lead to contextual bias. By using a likelihood ratio, juries could base their decisions on the most updated posterior odds ratio, which depends heavily on the prior odds (prior beliefs), and examiners could provide relevant observations without having to know all aspects of the case.

To address the concerns raised above and to suggest an alternative approach to categorical conclusions, the goal of this paper is to create a value that expresses the actual strength of supporting the two propositions on an absolute scale. We compute likelihood ratios based on the distribution of human expert judgment in error‐rate studies, which can be summarized using an ordered probit model, which, when combined with the ground truth for each pair and several additional steps described later, allows us to compute the likelihood ratio that represents the strength of support for each proposition for each pair. Because this approach relies on the ordered probit model, we term these values Ordered Probit Likelihood Ratios. To begin, we first describe the ordered probit model.

### 1.1 The ordered probit model

**FIGURE 1:**
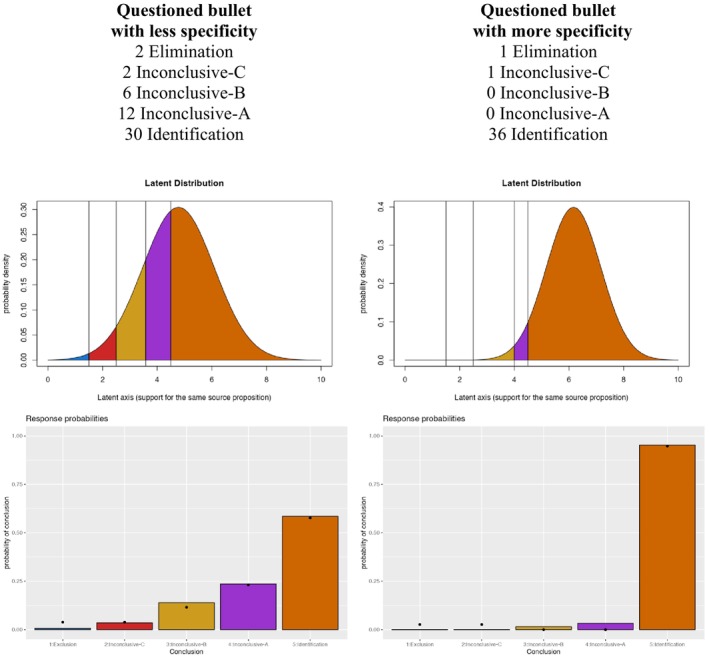
**Right Column:** Illustration of an ordered probit model applied to hypothetical firearm response probabilities (black dots in lower panel) with the best fitting parameters. Thirty examiners reached an Identification conclusion, with number for each conclusion shown above. **Left column:** Illustration of an ordered probit model applied to hypothetical firearm response probabilities (black dots in lower panel) with the best fitting parameters. Thirty‐six examiners reached an Identification conclusion, with a number for each conclusion shown above. [Correction added on 22 November 2024, after the first online publication: Figure 1 and its caption have been revised in this version.]

The ordered probit model summarizes the underlying distribution of location along the latent axis that leads to the observed collection of responses. Essentially, it provides a summary of the strength of support for the same source proposition as determined by the combined responses of all of the examiners who completed a comparison. Consider the two examples given in the two columns of Figure 1. The questioned bullet in the left column has responses that include all five conclusions, while the questioned bullet in the right column has mostly Identification conclusions. It would be inappropriate to try to average these responses or assign numbers to each category and then average because the responses are only on an ordinal scale. Instead, the ordered probit model summarizes these distributions of responses for each sample to create a numerical value that represents the typical strength of support for the same source proposition. As shown in Figure 1, the ordered probit model uses a latent axis that represents the amount of support for the same source propositions. We assume that at the end of each comparison, each examiner mentally ends up with a value along this latent axis that represents the amount of support for the same source proposition (or the relative support for the same‐ and different‐source propositions). We then assume that the collection of values across examiners along the latent axis can be summarized with a normal distribution for each pair. The model then assumes that the location along the latent axis is translated by examiners into one of the five conclusions through the application of four thresholds, such that if the latent value falls above the highest threshold, the examiner produces an Identification conclusion, and if the value falls below the lowest threshold, the examiner produces an Elimination conclusion. Values in between the outer two thresholds result in one of the three subdivisions of the inconclusive conclusion as determined by the two interior thresholds. The ordered probit model predicts the response frequency for each conclusion based on the area under the normal distribution between different thresholds, which are a reflection of the system‐wide behavior and do not reflect the thresholds of a single examiner.

The examples provided in Figure 1 illustrate how the model translates different distributions of examiner responses into the latent dimension. The left column of Figure 1 illustrates the model applied to a questioned bullet with less specificity and image detail, where a bare majority of experts reached an Identification decision. On the right column, we can see the model applied to a questioned bullet with more specificity and image detail, where a super‐majority of experts reached an Identification decision. The distribution of responses is shown in each column, and the lower graphs show the predicted response frequencies for each pair produced by the ordered probit model with the appropriate parameters for each questioned bullet. The ordered probit model assumes that the proportion of examiners who reach each conclusion is determined by the area under the normal distribution within each set of decision thresholds. For example, to produce more identification responses, the right column uses a normal distribution that is shifted to the right, which puts more area under the normal distribution to the right of the highest threshold (orange area). We have developed an interactive demonstration of the ordered probit model, and the reader is invited to explore different parameter settings to gain an intuition for the relation between the position of the normal distribution and the predicted distribution of verbal responses:

https://iupbsapps.shinyapps.io/OrderedProbitDemoFirearms/.

We use MCMC procedures (described below) to determine the most credible parameters for the ordered probit model, which summarize the support for the same source of proposition offered by each pair. The mean (μ) in the normal distribution corresponds to the typical level of support for the two propositions as indicated by the examiner responses, where higher μ values indicate greater support for the same source proposition. In the example illustrated in Figure 1 above, a pair with a super‐majority of Identification responses would have a greater μ value than a pair with a bare majority of Identification responses. The standard deviation σ reflects the consistency among examiners. We obtain the most credible values of μ and σ for each pair, along with the interior thresholds. Once we obtain the distribution of μ and σ parameters for each pair along with the two estimated thresholds, we can use these in combination with the ground truth (mated or nonmated) and two other assumptions (described in a later section) to calculate ordered probit likelihood ratios for individual bullet pairs. First, we describe an error rate study to illustrate how this approach would work on real‐world data.

### 1.2 Applications to error rate studies: Monson, Smith and Bajic (2022) [7]

Black Box studies have been introduced as a way to evaluate scientific validity in the comparison discipline [16]. In those studies, researchers treat the examiner as a “black box” in order to measure the accuracy of the system without detailed knowledge of the underlying processes. The examiner is asked to determine if the item(s) from the known and unknown sources originated from the same source after being shown an item(s) from an unknown origin. In order to decide if the sample of unknown origin is appropriate for comparison, forensic firearm and tool mark examiners start with a suitability check, ensuring that there are necessary markings to compare. When the sample is considered of value for purposes of comparison, experts look for class characteristics to make sure that the class characteristics are consistent. Finally, experts would look for individual characteristics and share their results employing three categories: no value, value for elimination alone, and value for individualization. The examiner comes to another categorical conclusion regarding the origin of the unknown if the item is considered appropriate for comparison, that is, value for elimination alone and value for individualization. Generally speaking, this conclusion falls into one of three general categories: identification, elimination, or inconclusive. The latter is subdivided into three categories in which there is either “some agreement of individual characteristics and all discernable class characteristics, but insufficient for identification,” “agreement of all discernable class characteristics without agreement or disagreement of individual characteristics due to an absence, insufficiency, or lack of reproducibility,” or “agreement of all discernable class characteristics and disagreement of individual characteristics, but insufficient for an elimination” [1].

In the current study, we are using data from Monson, Smith, and Bajic (2022) [7], who measured the performance of examiners in comparisons of both bullets and cartridge cases. The Monson study tested 173 examiners who volunteered through the AFTE website. For each Beretta firearm in the study, 700 test fires were created. For each non‐Beretta firearm in the study, 850 test fires were created for a total of 28,250 test fires. Every test packet contained 15 sets of cartridge cases and 15 sets of bullets that were mailed to the participants/ examiners in the study. A single questioned specimen was included in each comparison set, along with two known specimens that were fired from the same firearm and within the same sequence group of 50. Ten Beretta specimens and five Jimenez specimens were compared for cartridge cases, and ten Beretta specimens and five Ruger specimens were compared for bullets. Examiners were required to use the AFTE range of conclusions to determine whether each individual pair set was suitable, inconclusive (A, B, or C), identification, elimination, or unsuitability after examination under a comparison microscope. For more details about the study design, please refer to [7].

### 1.3 Ordered probit likelihood ratios from bullet comparisons

The process of calculating an ordered probit likelihood ratio for each pair in a database begins with the response distribution from examiners, and the right columns of Table 1 provide the response distribution for the Bullet data from the Monson Black Box study. Each row provides the number of examiners who reach each conclusion for each pair in the study. For example, in pair 6–6 shown in the left column of Figure 1, 30 examiners said Identification, 12 said Inconclusive‐A, 6 said Inconclusive‐B, 2 said Inconclusive‐C, and 2 said Elimination. Contrast this pair with pair G‐G, shown in the right column of Figure 1, which has 36 examiners said Identification, 0 said Inconclusive‐A, 0 said Inconclusive‐B, 1 said Inconclusive‐C, and 1 said Elimination. These two samples give very different support for the same source proposition, despite the fact that both were majority IDs. To fit the ordered probit model, we characterize the differences in support by assuming that there exists an underlying latent dimension that goes from the most support imaginable for the different source propositions to the most support imaginable for the same source proposition. Every pair results in a value along that dimension that is internal to each examiner. When we apply a set of decision thresholds to this latent dimension, we produce a predicted set of conclusions by all examiners who completed each comparison. The ordered probit model infers the underlying distribution of responses on the latent axis and summarizes it with a normal distribution with mean μ and standard deviation σ. To fit the ordered probit model to a set of data, we first need to establish the scale of the latent axis, much like 0° and 100° establish the scale for Celsius temperature measurements. Without loss of generality, in this case we set the threshold for the Elimination to 1.5 and the threshold for identification to 4.5 on the latent scale. Because the scale includes Elimination, Inconclusive‐c for insufficient for elimination, Inconclusive‐b for lack of reproducibility, Inconclusive‐a for insufficient for identification, and Identification, we need two inner decision thresholds that are estimated across all pairs in the dataset. In addition, each pair has its own estimated mean μ and σ for the normal distribution on the latent dimension, which characterizes its support for the same source proposition based on the examiner responses. The most credible parameters were found using a Monte Carlo Markov Chain estimation with the JAGS package [17]; [18] in R. Similarly to Busey and Coon [19], we used 500 initialization steps and 1000 adaptation steps, and a final chain of 60,000 steps thinned every 5 steps.

**TABLE 1:** Representative data from the Bullet data from Monson Error Rate investigation.

Pair ID	Mated	Mu	Sigma	LR	Elim	Inc‐C	Inc‐B	Inc‐A	ID	Majority ID
I‐Q	FALSE	0.55	1.47	0.04	14	3	2	0	0	FALSE
L‐T	FALSE	0.70	1.50	0.05	13	5	0	1	0	FALSE
V‐C	FALSE	0.93	1.38	0.05	13	4	3	0	0	FALSE
7–10	FALSE	1.08	1.46	0.05	10	5	1	1	0	FALSE
7–11	FALSE	1.23	0.82	0.06	11	8	0	0	0	FALSE
J‐R	FALSE	1.34	1.67	0.06	11	3	4	2	0	FALSE
Y‐G	FALSE	1.38	1.63	0.06	11	7	2	1	1	FALSE
Q‐Y	FALSE	1.51	1.56	0.07	11	4	5	2	0	FALSE
H‐P	FALSE	1.55	1.77	0.07	9	4	1	4	0	FALSE
N‐T	FALSE	1.61	1.60	0.07	8	1	6	1	0	FALSE
P‐X	FALSE	1.64	1.47	0.07	10	5	5	2	0	FALSE
G‐O	FALSE	1.73	1.62	0.08	7	4	2	3	0	FALSE
T‐B	FALSE	1.77	1.39	0.08	8	8	2	3	0	FALSE
M‐V	FALSE	1.78	1.30	0.08	7	5	5	1	0	FALSE
H‐Q	FALSE	1.80	1.41	0.08	8	5	5	2	0	FALSE
E‐L	FALSE	1.81	1.51	0.08	6	5	4	0	1	FALSE
V‐D	FALSE	1.86	1.31	0.09	7	8	5	0	1	FALSE
L‐U	FALSE	1.88	1.65	0.09	7	4	2	4	0	FALSE
3–7	FALSE	1.91	1.11	0.09	7	8	6	1	0	FALSE
Y‐F	FALSE	1.91	1.19	0.09	5	6	4	1	0	FALSE
G‐P	FALSE	1.94	1.19	0.09	7	6	7	1	0	FALSE
10–3	FALSE	1.96	1.47	0.09	8	2	8	2	0	FALSE
N‐W	FALSE	1.98	1.20	0.10	5	8	3	2	0	FALSE
N‐V	FALSE	2.05	1.37	0.10	6	6	4	3	0	FALSE
11–5	FALSE	2.08	1.48	0.10	10	4	11	2	1	FALSE
4–7	FALSE	2.10	0.88	0.11	3	7	6	0	0	FALSE
10–4	FALSE	2.15	1.20	0.11	9	5	13	2	0	FALSE
U‐C	FALSE	2.18	1.64	0.11	7	5	4	4	1	FALSE
P‐Y	FALSE	2.20	1.29	0.11	5	6	5	3	0	FALSE
E‐N	FALSE	2.26	1.40	0.12	5	8	4	3	1	FALSE
J‐S	FALSE	2.29	1.42	0.12	6	5	5	5	0	FALSE
O‐X	FALSE	2.31	1.28	0.13	4	5	5	3	0	FALSE
6–10	FALSE	2.34	1.33	0.13	5	4	9	1	1	FALSE
7–1	FALSE	2.43	0.98	0.14	3	10	9	3	0	FALSE
1–5	FALSE	2.47	0.95	0.15	3	7	11	2	0	FALSE
Q‐Z	FALSE	2.53	1.33	0.16	4	6	4	6	0	FALSE
3–8	FALSE	2.63	1.11	0.17	3	5	9	4	0	FALSE
5–9	FALSE	2.67	0.91	0.18	2	2	12	1	0	FALSE
9–2	FALSE	2.68	0.96	0.18	2	3	11	2	0	FALSE
4–9	FALSE	2.81	0.84	0.21	1	3	12	2	0	FALSE
5–10	FALSE	2.87	0.89	0.22	1	3	11	3	0	FALSE
9–3	FALSE	2.95	1.00	0.25	3	0	18	3	1	FALSE
9–9	TRUE	4.19	1.31	1.54	1	1	14	11	18	FALSE
8–8	TRUE	4.35	1.77	2.04	2	3	18	4	26	FALSE
6–6	TRUE	4.88	1.75	**5.43**	2	2	6	12	30	TRUE
5–5	TRUE	4.93	1.63	**5.93**	1	1	10	9	32	TRUE
1–1	TRUE	5.60	1.66	**22.14**	0	1	6	6	39	TRUE
H‐H	TRUE	5.80	2.44	**33.07**	2	2	5	2	30	TRUE
S‐S	TRUE	6.22	1.79	**79.03**	0	0	3	1	22	TRUE
Y‐Y	TRUE	6.41	1.58	**115.66**	0	0	1	2	24	TRUE
B‐B	TRUE	6.46	1.71	**128.60**	0	1	0	4	35	TRUE
U–U	TRUE	6.61	1.95	**175.78**	0	0	4	0	29	TRUE
E–E	TRUE	6.83	2.71	**279.25**	2	1	0	1	23	TRUE
D‐D	TRUE	6.86	2.01	**300.87**	0	1	1	1	26	TRUE
P–P	TRUE	6.99	1.89	**391.37**	0	1	0	2	32	TRUE
A‐A	TRUE	7.23	2.03	**666.58**	0	1	1	1	36	TRUE
W‐W	TRUE	7.35	2.39	**864.31**	1	0	2	0	30	TRUE
F‐F	TRUE	7.63	2.28	**1567.90**	1	0	0	1	28	TRUE
V‐V	TRUE	8.12	2.57	**4741.83**	1	1	0	0	35	TRUE
G‐G	TRUE	8.15	2.57	**5106.26**	1	1	0	0	36	TRUE
T–T	TRUE	9.48	1.33	**117562.99**	0	0	0	0	26	TRUE

*Note*: We calculated the μ and σ values using the ordered probit model and sorted the pairs from the lowest μ to the highest μ. The numbers on the right side of the table represent the number of examiners who responded with an Individualization (ID), Elimination (Elim), or Inconclusive (Inc‐C, Inc‐B, and Inc‐A). Each pair's ground truth is indicated by the column “Mated” with False referring to nonmated pairs and True referring to mated pairs. Bold likelihood ratio values are those pairs in which examiners gave more Identification decisions than all other responses, which reflects those comparisons that might be considered casework‐like quality. The full table can be found in the Supplementary Information as Table S1.

**FIGURE 2:**
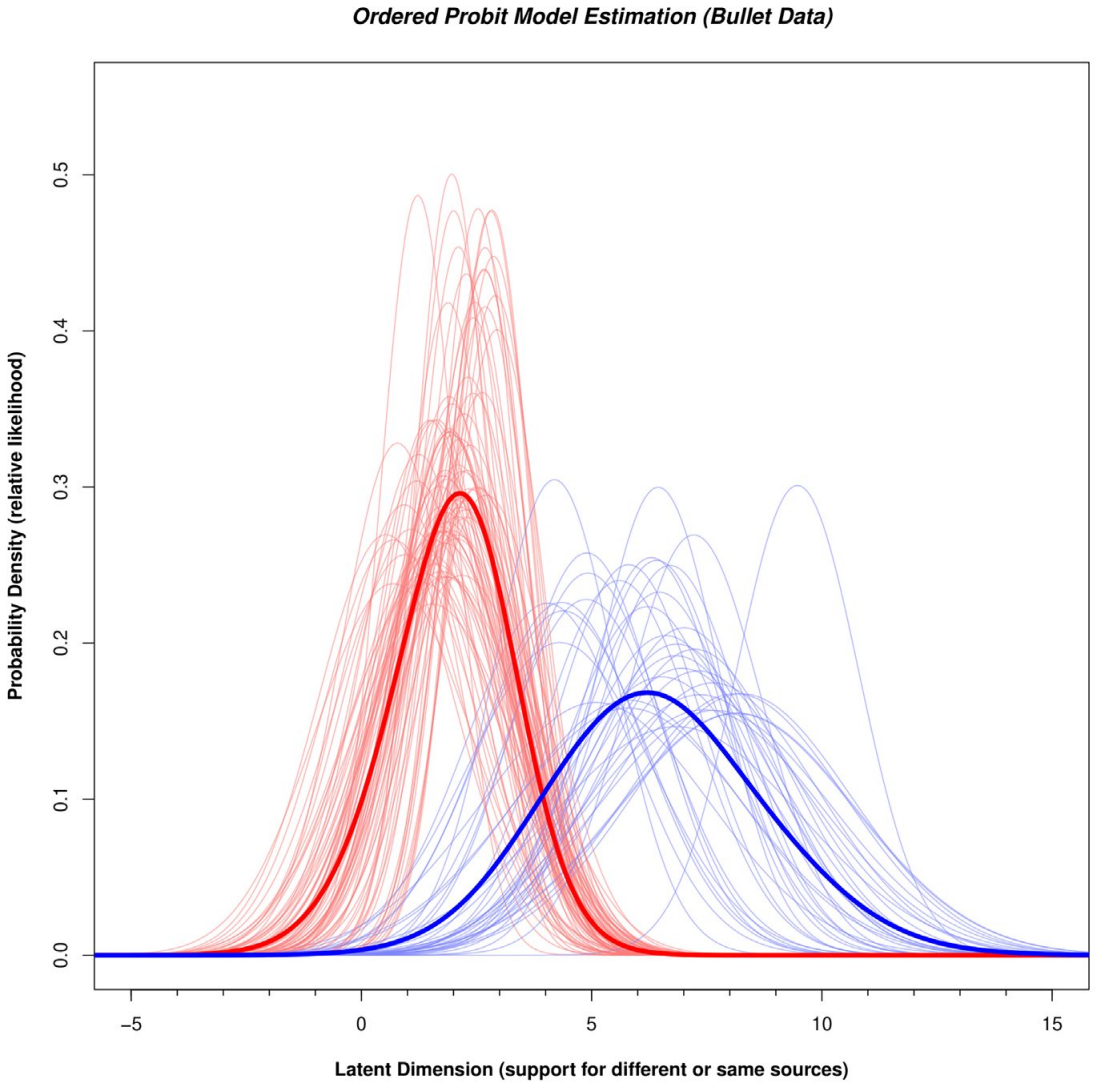
Relative likelihood of observing a given latent value for each mated (light blue curves) or nonmated (light red curves) comparison for the Bullet data (Monson, 2022). The parameters for each normal distribution were derived from the ordered probit model fit to all five conclusions for each comparison. The thick red curve corresponds to the sum of light red curves. It represents the relative likelihood of observing any nonmated comparison at each value of the latent axis. The thick blue curve represents the relative likelihood of observing any mated comparison at each value of the latent axis.

Figure 2 illustrates all the normal distributions defined by their parameters μ and σ for each bullet pair. Every light red curve represents the latent distribution of a nonmated pair, while every light blue curve corresponds to the latent distribution of a mated pair. The variation in location along the latent axis for the light blue and light red curves shows that different bullet pairs offer varying degrees of support for the same source proposition. The height of each curve represents the probability density of the normal distribution, which is the likelihood of an expert reaching that particular latent value given that bullet pair was compared.

**FIGURE 3:**
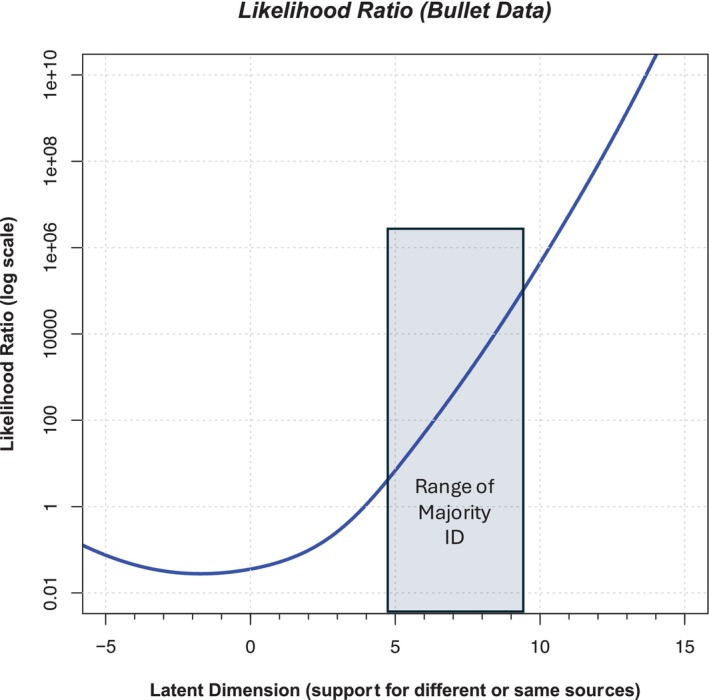
Likelihood ratio values for different values along the latent axis for the Bullet data (Monson, 2022). The y‐axis is plotted on a log(10) axis. The log of the likelihood ratio can be observed directly as the difference between the thick blue and thick red curves in Figure 2. The blue region illustrates the approximate range of bullet pairs with majority ID decisions. [Correction added on 18 Nov 2024, after the first online publication: Figure 3 has been updated in this version.]

There are two additional steps required to compute likelihood ratios from the output of the ordered probit model: The first step is to generate overall curves that summarize all of the mated and all of the nonmated curves. The thin curves in Figure 2 reflect the probability of the observation (value along the latent axis) given that particular pair was presented. However, what we really want is a likelihood ratio that is computed relative to the same‐ and different‐source propositions, not which pair was presented. Such a likelihood ratio is the probability of the observation (the location along the latent axis) given *any nonmated or any mated pair*. To compute the probability of a particular latent value given a mated pair, we assume that the pairs are independent, and we use the “or” rule in probability to add all the mated normal distributions together and renormalize to an area of 1.0. This creates the probability of each latent value given any mated pair and is shown as the thick blue curve in Figure 2. We repeat this process for the nonmated pairs to produce the probability density for any nonmated pair, which is shown as the thick red curve in Figure 2. The likelihood ratio is the ratio of the two thick curves in Figure 2 at every value along the latent axis. Figure 3 illustrates the ordered probit likelihood ratios computed for every value along the latent axis and illustrates that higher ordered probit likelihood ratios are associated with larger μ values. This relation is to be expected: As more examiners make an Identification, the support for the same source proposition should increase. Note that this relation is fairly linear when the ordered probit likelihood ratios are plotted on a log axis, which did not have to occur but is entirely reasonable. The second step computes ordered probit likelihood ratios for individual pairs. In the Monson study, we can use the most credible μ value for each pair in the database as the estimate of the typical strength of support offered by that pair for the two propositions. We then consult the curve in Figure 3 at the μ value for that pair to determine the ordered probit likelihood ratio associated with that pair.

Together, these two assumptions create ordered probit likelihood ratios for each bullet pair in the database. Table 1 provides the response distributions for the Bullet data from the Monson Black Box study for half of the pairs, and the full table is found in the Supplemental Material as Table S1. The rows are sorted by the μ value, with nonmated pairs that received mostly Elimination responses at the top. Pairs lower down have more Inconclusive responses, and pairs toward the bottom have more ID responses. Following this trend, the lower rows tend to have larger *μ* values. A likelihood ratio of 1 implies equal support for the two propositions. Values larger than 1 correspond to more support for the same source of proposition, and values less than 1 correspond to more support for different sources of proposition.

We might consider a bullet pair that reached a majority of Identification responses as “actionable casework” and are likely to be reported out to a forensic service provider partner such as a prosecutor or jury. The bold ordered probit likelihood ratio values in Table 1 are the pairs in which examiners gave more Identification decisions than all other responses. These ordered probit likelihood ratios range from 5 to 100,000 and are associated with μ values ranging from 4.88 to 9.45. [Correction added on 16 Nov 2024, after the first online publication: In the preceding sentence, the value “4..38” has been changed to “4.88” in this version.] We explore the implications of these values in the discussion below, but first we analyze cartridge cases from the Monson et al. study. Figures [Link], [Link], [Link], [Link], [Link], [Link], [Link], [Link], [Link] provide further analysis and insights into the choice of model parameters.

### 1.4 Ordered probit likelihood ratios from cartridge case comparisons

**FIGURE 4:**
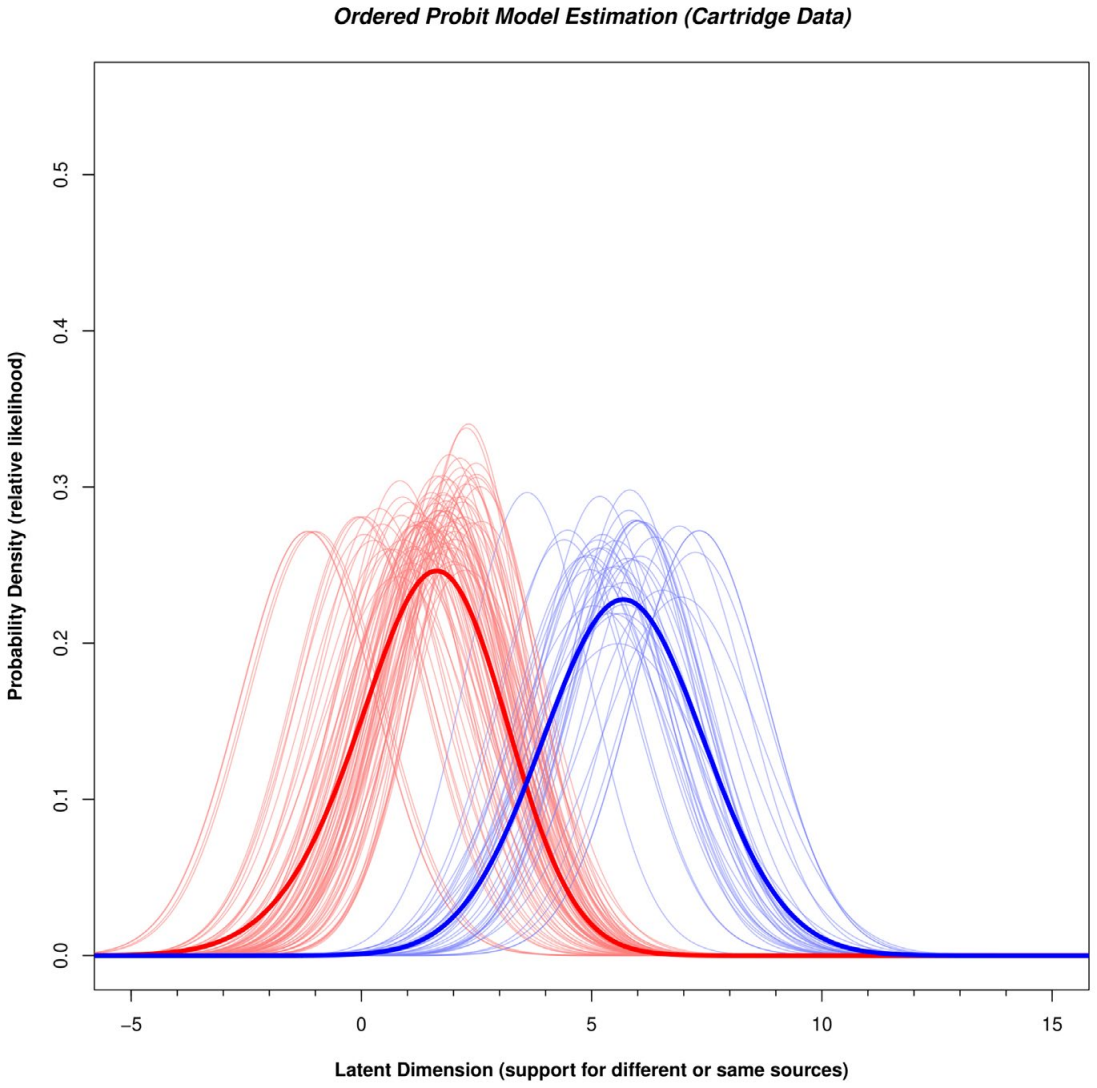
Relative likelihood of observing a given latent value for each mated (light blue curves) or nonmated (light red curves) comparison for the Cartridge data (Monson, 2022). The parameters for each normal distribution were derived from the ordered probit model fit to all five conclusions for each comparison. The thick red curve corresponds to the sum of light red curves. It represents the relative likelihood of observing any nonmated comparison at each value of the latent axis. The thick blue curve represents the relative likelihood of observing any mated comparison at each value of the latent axis.

**FIGURE 5:**
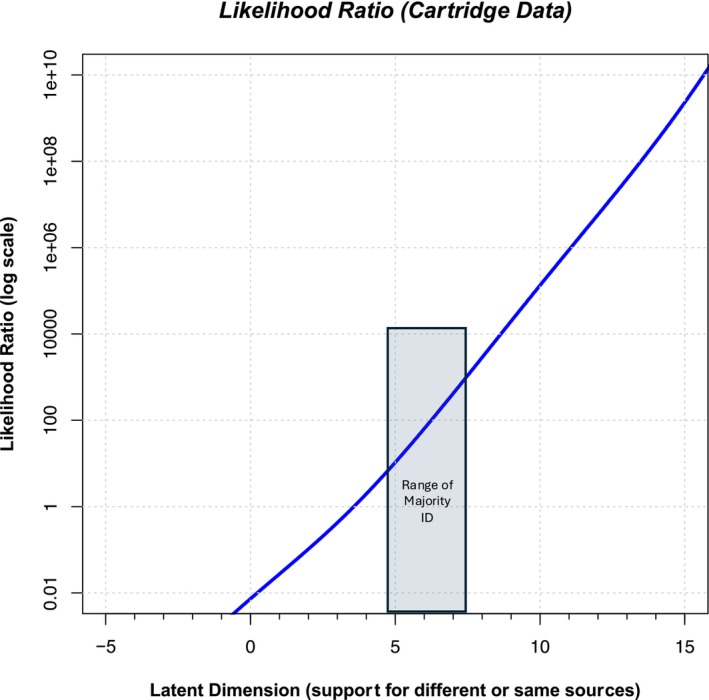
Likelihood ratio values for different values along the latent axis for the cartridge data (Monson, 2022). The y‐axis is plotted on a log(10) axis. The log of the likelihood ratio can be observed directly as the difference between the thick blue and thick red curves in Figure 4. The blue region illustrates the approximate range of cartridge pairs with majority ID decisions. [Correction added on 18 Nov 2024, after the first online publication: Figure 5 has been updated in this version.]

We applied the ordered probit model to Cartridge data from the Monson study [7] using the same procedures as used with the bullets. Table 2 provides the response distributions for each cartridge pair in the database, along with the parameters of the ordered probit model and the ordered probit likelihood ratios for each image pair. The relative likelihood of observing a specific latent value for each pair of mated (light blue curves) or nonmated (light red curves) cartridges is displayed in Figure 4. We are not interested in the individual curves, but in whether any pair was presented. So we consider all light curves to be independent and add them to produce the red and blue thick curves. The thick blue curve corresponds to the probability of each latent value given any mated pair, and the thick red curve corresponds to the probability of each latent value given any nonmated pair. Plotted in Figure 5 is the ordered probit likelihood ratio, the ratio of thick blue to thick red curve values at each point along the latent axis in Figure 4. The values are plotted on a log ordinate axis. By determining the height of the red and blue curves in Figure 4 at each μ value, we can compute an ordered probit likelihood ratio for each cartridge pair in the dataset. The μ values for casework‐like quality that we identified in Table 2 correspond to ordered probit likelihood ratios ranging from 8 to 810. These values as somewhat lower than the ordered probit likelihood ratios from Bullet data, which can result from lower cartridge quality examined in the Monson study.

**TABLE 2:** Representative data from the cartridge data from the Monson Black Box investigation [7].

Pair ID	Mated	Mu	Sigma	LR	Elim	Inc‐C	Inc‐B	Inc‐A	ID	Majority ID
7–1	FALSE	−1.16	1.47	0.00	25	1	0	0	0	FALSE
7–11	FALSE	−1.05	1.47	0.00	21	1	0	0	0	FALSE
F‐O	FALSE	−0.08	1.42	0.01	17	3	0	0	0	FALSE
H‐O	FALSE	0.06	1.48	0.01	15	2	1	0	0	FALSE
O‐X	FALSE	0.26	1.50	0.01	16	2	2	0	0	FALSE
N‐V	FALSE	0.45	1.44	0.01	12	3	1	0	0	FALSE
11–5	FALSE	0.72	1.54	0.02	19	4	3	1	0	FALSE
G‐N	FALSE	0.83	1.31	0.02	10	6	0	0	0	FALSE
8–1	FALSE	0.89	1.36	0.02	14	6	2	0	0	FALSE
K‐R	FALSE	1.04	1.37	0.03	10	5	2	0	0	FALSE
H‐Q	FALSE	1.08	1.59	0.03	13	1	5	1	0	FALSE
V‐A	FALSE	1.15	1.45	0.03	9	5	1	1	0	FALSE
Q‐Z	FALSE	1.20	1.45	0.04	11	3	5	0	0	FALSE
M‐V	FALSE	1.23	1.44	0.04	9	3	4	0	0	FALSE
L‐S	FALSE	1.37	1.44	0.05	8	5	2	1	0	FALSE
I‐P	FALSE	1.41	1.44	0.05	9	5	3	1	0	FALSE
I‐R	FALSE	1.44	1.57	0.05	10	2	4	2	0	FALSE
J‐R	FALSE	1.50	1.42	0.05	8	5	3	1	0	FALSE
C‐J	FALSE	1.51	1.48	0.05	8	3	4	1	0	FALSE
10–4	FALSE	1.61	1.50	0.06	12	9	3	2	1	FALSE
D‐M	FALSE	1.64	1.30	0.06	9	9	4	1	0	FALSE
4–9	FALSE	1.65	1.55	0.07	13	5	5	4	0	FALSE
F‐M	FALSE	1.68	1.40	0.07	7	5	4	1	0	FALSE
L‐T	FALSE	1.76	1.30	0.07	5	8	2	1	0	FALSE
1–4	FALSE	1.77	1.35	0.08	7	4	7	0	0	FALSE
V‐C	FALSE	1.79	1.35	0.08	6	6	4	1	0	FALSE
U‐C	FALSE	1.84	1.37	0.08	7	5	6	1	0	FALSE
3–8	FALSE	1.84	1.51	0.08	11	4	9	1	1	FALSE
J‐Q	FALSE	1.89	1.45	0.09	7	6	3	3	0	FALSE
W‐D	FALSE	1.92	1.39	0.09	6	6	4	2	0	FALSE
5–10	FALSE	1.98	1.53	0.10	9	6	5	3	1	FALSE
T‐A	FALSE	2.04	1.50	0.11	6	3	4	3	0	FALSE
I‐Q	FALSE	2.05	1.59	0.11	7	2	5	2	1	FALSE
Y‐G	FALSE	2.13	1.42	0.12	5	7	2	4	0	FALSE
6–10	FALSE	2.16	1.27	0.13	4	8	5	2	0	FALSE
AA‐G	FALSE	2.20	1.36	0.14	4	5	5	2	0	FALSE
4–8	FALSE	2.23	1.42	0.14	5	7	5	2	1	FALSE
5–9	FALSE	2.28	1.18	0.15	4	7	10	1	0	FALSE
V‐D	FALSE	2.33	1.17	0.16	3	7	9	1	0	FALSE
Z‐H	FALSE	2.49	1.30	0.20	3	4	10	0	1	FALSE
P‐X	FALSE	2.50	1.30	0.21	2	5	8	0	1	FALSE
C‐L	FALSE	2.61	1.43	0.24	6	2	8	7	0	FALSE
6–6	TRUE	4.40	1.50	3.81	2	1	15	11	26	FALSE
Z‐Z	TRUE	4.86	1.56	**8.34**	1	0	6	5	18	TRUE
5–5	TRUE	4.97	1.62	**10.03**	1	2	7	8	30	TRUE
11–11	TRUE	5.13	1.53	**13.32**	1	2	2	13	33	TRUE
M‐M	TRUE	5.18	1.36	**14.50**	0	0	3	7	20	TRUE
D‐D	TRUE	5.24	1.48	**16.06**	0	0	6	4	23	TRUE
10–10	TRUE	5.41	1.61	**21.94**	1	1	5	9	41	TRUE
W‐W	TRUE	5.52	1.50	**26.43**	0	0	4	3	22	TRUE
7–7	TRUE	5.57	2.00	**29.39**	3	2	4	3	37	TRUE
K–K	TRUE	5.70	1.67	**36.96**	1	0	2	3	22	TRUE
L‐L	TRUE	5.79	1.57	**43.39**	0	0	4	1	22	TRUE
AA‐AA	TRUE	5.83	1.34	**46.76**	0	0	0	6	27	TRUE
Q‐Q	TRUE	5.93	1.43	**56.55**	0	0	1	4	25	TRUE
U–U	TRUE	6.00	1.43	**63.44**	0	0	1	4	27	TRUE
C‐C	TRUE	6.06	1.56	**71.19**	0	1	1	4	33	TRUE
Y‐Y	TRUE	6.37	1.49	**128.04**	0	0	1	2	26	TRUE
F‐F	TRUE	6.90	1.45	**349.09**	0	0	0	2	36	TRUE
T–T	TRUE	7.26	1.55	**695.59**	0	0	1	0	30	TRUE
A‐A	TRUE	7.34	1.47	**803.18**	0	0	0	1	33	TRUE

*Note*: We calculated the μ and σ values using the ordered probit model and sorted the pairs from the lowest μ to the highest μ. The numbers on the right side of the table represent the number of examiners who responded with an Individualization (ID), Elimination (Elim), or Inconclusive (Inc‐C, Inc‐B, and Inc‐A). Each pair's ground truth is indicated by the column “Mated” with False referring to nonmated pairs and True referring to mated pairs. Bold likelihood ratio values are those pairs in which examiners gave more Identification decisions than all other responses, which reflects if a comparison might be considered casework‐like quality. The full table can be found in Table S2.

From Tables 1 and 2, we observe that the transition between nonmated and mated pairs corresponds to the transition of likelihood ratios from less than one to greater than one. In addition, the separation between mated and nonmated pairs is abrupt, with no nonmated pairs above mated pairs and vice versa. Thus, despite substantial overlap between the blue and red curves in Figure 1, when the data from multiple experts is combined, this leads to a clear separation between mated and nonmated pairs.

### 1.5 Comparison with other likelihood ratios

The ordered probit likelihood ratios presented in the present analysis depend on the assumptions of the ordered probit model and might be sensitive to these assumptions (especially the normal distribution). In the Supplementary Information section—Data S1, we perform a sensitivity analysis that addresses the robustness of these assumptions, including a t‐distribution instead of a normal distribution, wider priors on the μ values, and turning off shrinkage on the normal distribution standard deviation values. None of these changes to the assumptions underlying the ordered probit model systematically increased the likelihood ratios across all values of μ. Indeed, many of these changes increased the likelihood ratios for comparisons with larger values of μ at the expense of likelihood ratios for smaller values of μ, which is arguably worse than the original model because small likelihood ratios are of most concern when it comes to evidence interpretation at the weaker end of the scale. Overall, these sensitivity analyses demonstrate that the modest ordered probit likelihood ratios reported in this paper are not a result of particular assumptions or choices of parameter values for prior distributions.

There is another way to compute likelihood ratios that does not depend on the assumptions of the ordered probit model. Recall that the likelihood ratio is the ratio of the relative probability of the observations given two propositions. If we consider the Identification conclusion as an observation, we can calculate an overall likelihood ratio for both bullets and cartridges by computing the following ratio:

$$\mathrm{LR}=\frac{p\left(\text{Identification}|\text{mated}\right)}{p\left(\text{Identification}|\text{non mated}\right)}$$

The numerator for this equation is the correct identification rate, which is 0.77 for bullets and 0.74 for cartridges. The denominator is the erroneous identification rate, which is 0.0079 for bullets and 0.0080 for cartridges. This gives an overall likelihood ratio for bullet evidence of 97 and a likelihood ratio of 92 for cartridges. These values are entirely compatible with the likelihood ratio values produced by the ordered probit model, which ranged from less than 10 to around the thousands with many in the hundreds (see Tables 1 and 2). Given this alternative way of computing likelihood ratios, we do not believe that the ordered probit model underestimates the range of likelihood ratios for typical firearms casework and instead provides values that are consistent with other ways to estimate the strength of the observations.

One might ask why the ordered probit likelihood ratios are so modest in Tables 1 and 2 given the values of 10^4^ to 10^10^ reported by Roberge, Beauchamp, and Lévesque [20], who use statistical comparisons of bullet and cartridge evidence to construct likelihood ratios. In the present approach, we are not evaluating the evidentiary value of bullets and cartridge cases; we are evaluating the evidentiary value of the observations on the bullets and cartridge cases. Thus, our method describes the evidentiary value of human testimony that is based on personal experience and observations, and our approach would apply to similar situations where human observers rely on computer‐based tools to augment their conclusions. In such a setting, we could provide a likelihood ratio of the entire system rather than just one software tool.

### 1.6 Interpreting likelihood ratios

It is important to understand the range of likelihood ratios associated with comparisons for which a majority of examiners reached an Identification conclusion.

Tables 1 and 2 list the ordered probit likelihood ratios for different bullet and cartridge case pairs; however, those values need to be read within a specific context. For example, consider Pair 3–3 in.

Table 1: While 32 examiners said ID, there were still 15 who said Inconclusive and 6 who said Elimination, which resulted in an ordered probit likelihood ratio of 8.05. In court, when evaluating the same source proposition, juries and judges will have to incorporate the likelihood ratio value with the other evidence in the case. Bayesian updating involves updating prior beliefs about the support for the same source proposition based on new observations presented during the trial. This process allows for the integration of prior knowledge and the strength of the new evidence, leading to a more accurate assessment of the support for the same source proposition. Bayesian updating follows Bayes' theorem, which mathematically describes how to update the prior odds based on the new observation. The theorem states that the posterior odds of an event, given the observation, are proportional to the product of the prior odds and the likelihood ratio. After applying Bayes' theorem, we obtain the posterior odds, which represent our updated belief about the relative likelihood of the two propositions. The posterior odds serve as the updated prior odds for subsequent iterations of Bayesian updating. For example, prior to hearing a firearm likelihood ratio, a jury might believe that the support for the same source proposition is twice as likely as the support for a different source proposition (based on some evidence like eyewitness testimony). If a likelihood ratio from firearms evidence of 1000 is presented and the evidence is seen as credible and probative, a Bayesian update rule would multiply 2 times 1000 to obtain a posterior odds of 2000. Although this operation is straightforward, whether jurors can use the likelihood ratios appropriately is an open question because there is a tendency to combine evidence using an additive mechanism that is consistent with the scale of justice model for evidence combination [21]. This remains an active area of research in the field, although likelihood ratios are routinely presented in court in DNA analyses.

We view likelihood ratios as an alternative to the posterior predictive value approach. It is a measure of belief change, whereas a posterior predictive value asks the question of how likely you are to be wrong once you have made a conclusion. Our approach explicitly measures likelihood ratios for each comparison, while a posterior predictive value applies to all Identification decisions regardless of the complexity of the comparison or its borderline nature. One of the challenges of an expert making a posterior is that it requires the forensic examiner to know the priors, including other information and evidence that is outside the available case information.

### 1.7 Calibrating conclusion scales

The current articulation scales used by firearms examiners have not been calibrated against the actual strength of the firearm evidence, except indirectly through error rate studies. For example, what likelihood ratio is implied by the term Identification (ignoring for a moment that one is a belief change and the other is a posterior conclusion)? The Firearms and Toolmark Subcommittee of the Organization of Scientific Area Committees (OSAC) has produced a draft standard in which they associated Identification with “Extremely Strong Support for Common Source” [22]. In the verbal equivalency scale provided by the Scientific Working Group on DNA Analysis Methods [23], “very strong support” is associated with a likelihood ratio of over a million, implying that “extremely strong support” would require likelihood ratios of more than a million. Busey and Klutzke [24] demonstrated that both examiners and laypersons interpreted “identification” as providing more support for the same source position than a likelihood ratio of 100,000. One tempting suggestion is that DNA might continue to use their existing scale while firearms use another, which is a suggestion explicitly *rejected* by Marquis, Biedermann [2]. Table 3 presents the verbal equivalency scale developed by the Association of Forensic Science Providers. In Tables 1 and 2, pairs where examiners made more Identification decisions than any other responses showed ordered probit likelihood ratios as low as 5 for bullet data and 8 for cartridge data. Additionally, our ordered probit model suggests that the evidentiary strength of the term “Identification” is as low as 2.65 for Bullets and 4.49 for cartridges, which is far from the high likelihood ratios expected to be associated with the term “Identification. This suggests that the term “Identification” may not align with the actual strength of the evidence given the verbal equivalency scales described above and may be miscalibrated by more than five orders of magnitude.

**TABLE 3:** Relation between the likelihood ratios ranges and the corresponding verbal expressions developed by the Association of Forensic Science Providers [28].

Likelihood ratio	Verbal communication
10,000–1,000,000	This support is qualified as *very strong*
1000–10,000	This support is qualified as *strong*
100–1000	This support is qualified as *moderately strong*
10–100	This support is qualified as *moderate*
1–10	This support is qualified as *weak*

### 1.8 Implication for casework and criminal justice partners

The present work informs several ongoing debates in the firearms community. First, various organizations have suggested alternative articulation terms (or redefining existing ones) without sound scientific support for the terms or acknowledging the problem of using definitive conclusions. Second, there is a robust and ongoing debate about the term Inconclusive and whether it represents a third ground truth or how such conclusions should be reported or interpreted [25, 26]. In our view, likelihood ratios solve both problems because they are automatically calibrated to the strength of the evidence (they *are* the strength of the evidence), and an inconclusive observation is simply a likelihood ratio near 1.0.

One interim step would be to calibrate the articulation of the conclusions to the actual strength of support for the same source of proposition. This might require examiners to become more cautious in cases where examiners disagree, which our study demonstrates tends to be associated with lower ordered probit likelihood ratios. Part of the problem with current practices is that criminal justice partners are expecting examiners to provide the answer to the question “Did this gun fire this bullet?” However, making a decision is complicated by the fact that examiners typically have no knowledge of the prior likelihood of the gun firing the bullet because they may not understand how the evidence will be used in court. Instead, we believe that examiners should communicate the strength of support of their responses for the two propositions, which is a balanced approach [27]. Likelihood ratios serve this goal, and the present approach leverages human expertise to produce numerical values that represent the relative support for the two propositions. Verbal equivalency scales can provide guidance on how a forensic service provider partner should interpret the result. Importantly, the likelihood ratio is a measure of evidential strength that can be used to update your belief, and it is not a decision on the propositions.

How might this technique be used in casework? When an examiner has reached a response that represents the amount of support for the same source proposition, traditionally, they would label this with a verbal term such as Identification. An alternative method would create likelihood ratios for casework by comparing the amount of support offered by that pair against six or so benchmark pairs for which the ground truth and likelihood ratios were known. In practice, it might work like this: imagine a visualization with six benchmark pairs of bullets that were placed left to right on the screen depending on their amount of support for the same source proposition. An examiner could look at each pair and place their casework on the screen such that the pairs on the left would have less support for the same source proposition than the casework pair, and those on the right would have more support for the same source proposition than the casework pair. Based on interpolation of the relative positions of the pairs on the screen and the known likelihood ratios for the six benchmark pairs, we could compute a likelihood ratio for the casework comparison.

This method still relies on subjective comparisons of strength of support, but it has the advantage of grounding the casework strength of support in numerical likelihood ratios. It is true that an examiner could overestimate the strength of support for their particular casework, which would inflate the likelihood ratio. However, this is true with a definitive conclusion too, where some examiners are probably overusing the term Identification.

We are aware that this approach does not answer all the concerns about likelihood ratios and how they should be communicated to the fact finders. Implementing likelihood ratios would require changes to proficiency tests and verification steps, although DNA has shown that these are not insurmountable problems. We hope that this work can start a conversation among the community for how best to implement strength of support reporting, as the current definitive conclusion scale is not always calibrated against the strength of the evidence. This conversation will have to be a cross‐discipline to ensure that all parties are using terms in similar ways.

We and others view the role of forensic practitioners as making observations and communicating the evidential strength of those observations based on their interpretation, rather than deciding on the propositions. The decisions should be made by the fact finders, who have access to all of the relevant information. By shifting the focus from reporting categorical opinions to making observations, we encourage examiners to report the result of their interpretation as a likelihood ratio or a similar expression of strength of support.

## CONFLICT OF INTEREST STATEMENT

The authors have no competing interests to declare.

## PUBLIC SIGNIFICANCE STATEMENTS

This study challenges the verbal scale used by forensic examiners when communicating the strength of their conclusions, highlighting potential overstatements of the strength of the evidence. By introducing likelihood ratios as a quantitative measure, our research offers a more accurate representation of the strength of the evidence. This method has the potential to enhance communication with criminal justice partners and encourage examiners to shift their focus from reporting categorical opinions to making observations that express the amount of support for the same source proposition.

## Supporting information


Figure S1.

Figure S2.

Figure S3.

Figure S4.

Figure S5.

Figure S6.

Figure S7.

Figure S8.

Figure S9.

Table S1.

Table S2.

Data S1.

## Data Availability

The analyses generated for this study are available on Open Science Framework on the following link: https://osf.io/3ybhx/?view_only=c51dcb58608c432db13e5ea04b90585f.
