# Supplementary figures and images for: Quantifying the strength of firearms comparisons based on error rate studies
Source: J Forensic Sci. 2024 Oct 30;70(1):84–97. doi: 10.1111/1556-4029.15646 (PMC11693517; doi:10.1111/1556-4029.15646)

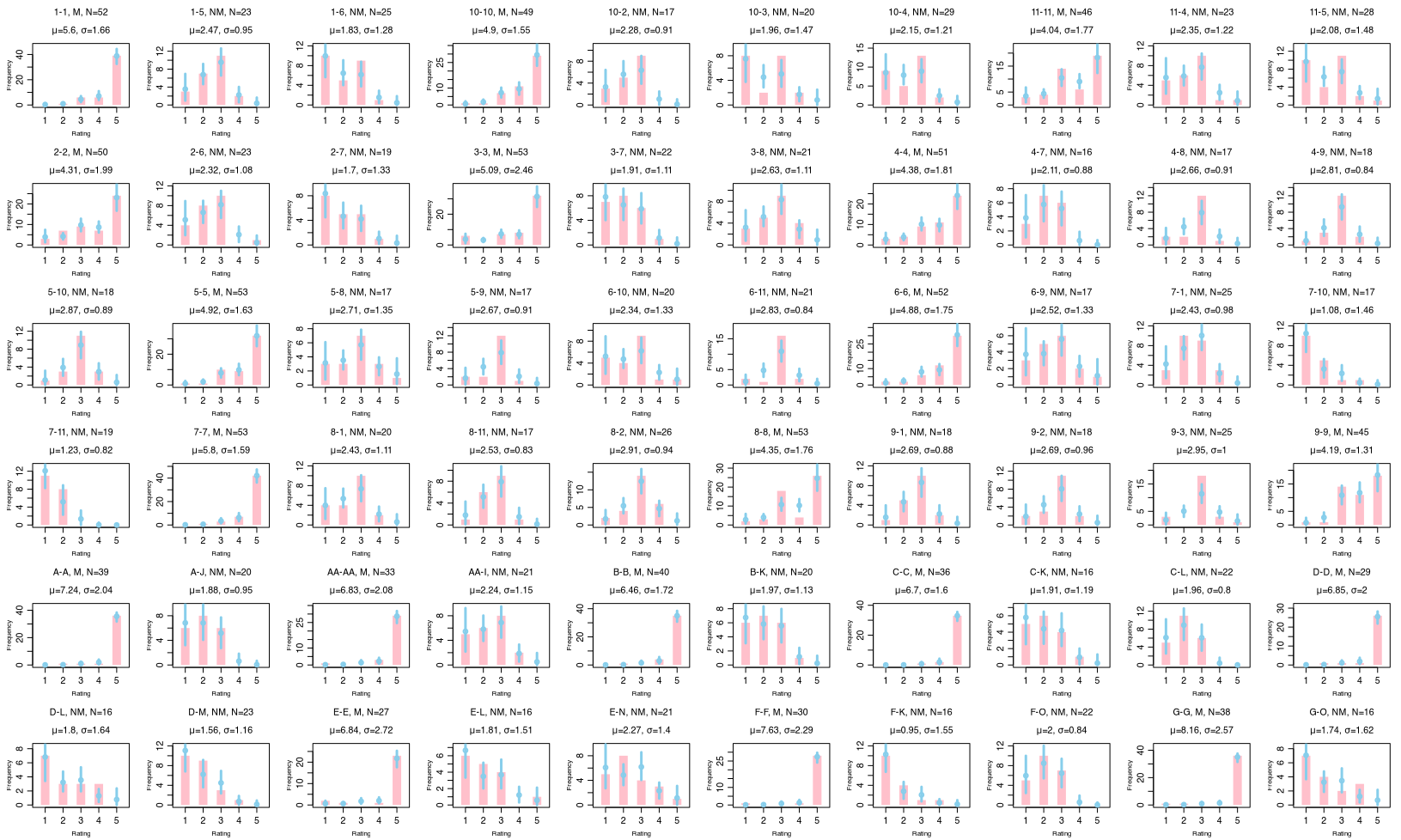

Supplement: Supplementary file 1 — Figure S1. [file JFO-70-84-s007.png]

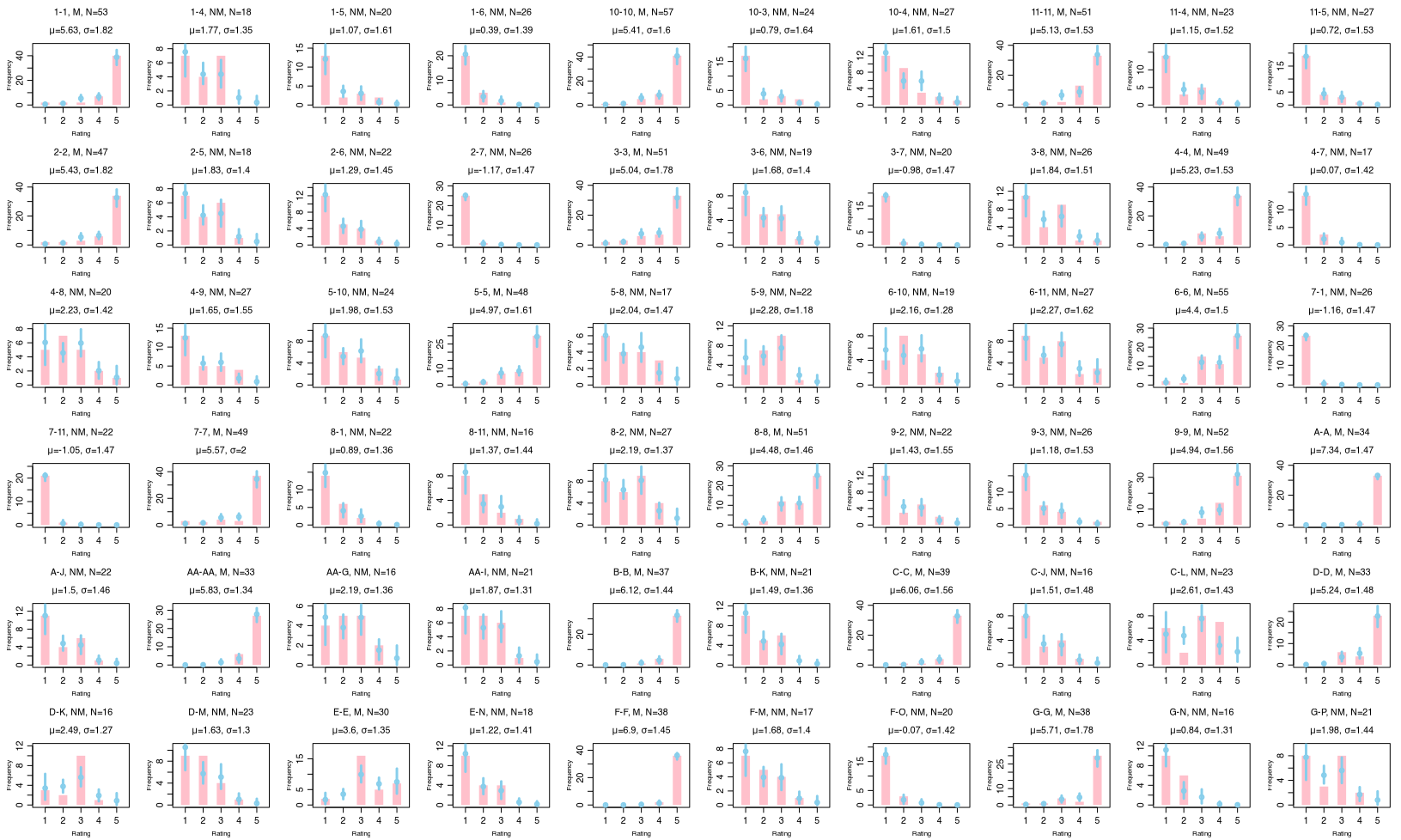

Supplement: Supplementary file 2 — Figure S2. [file JFO-70-84-s006.png]

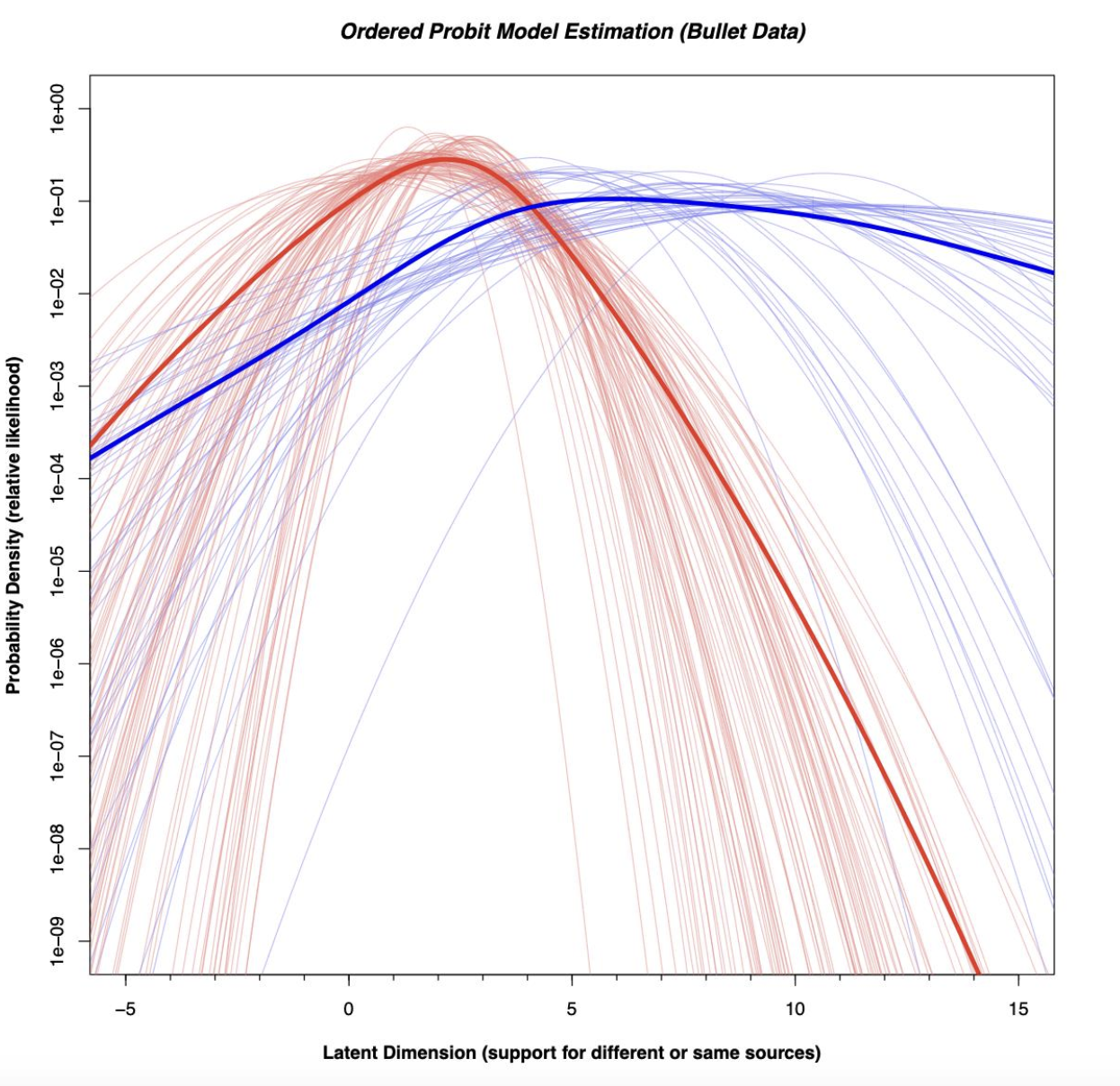

Supplement: Supplementary file 3 — Figure S3. [file JFO-70-84-s004.png]

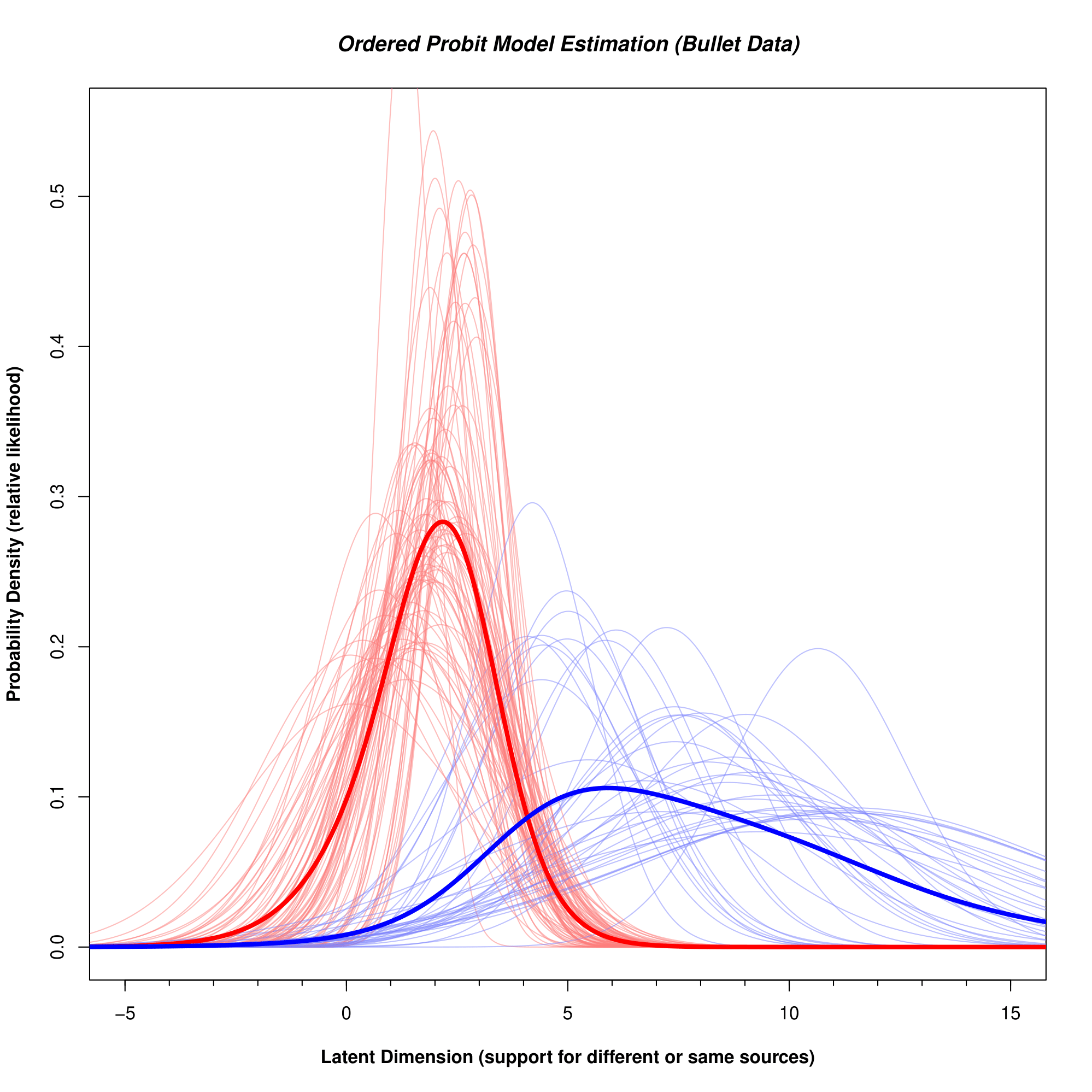

Supplement: Supplementary file 4 — Figure S4. [file JFO-70-84-s002.png]

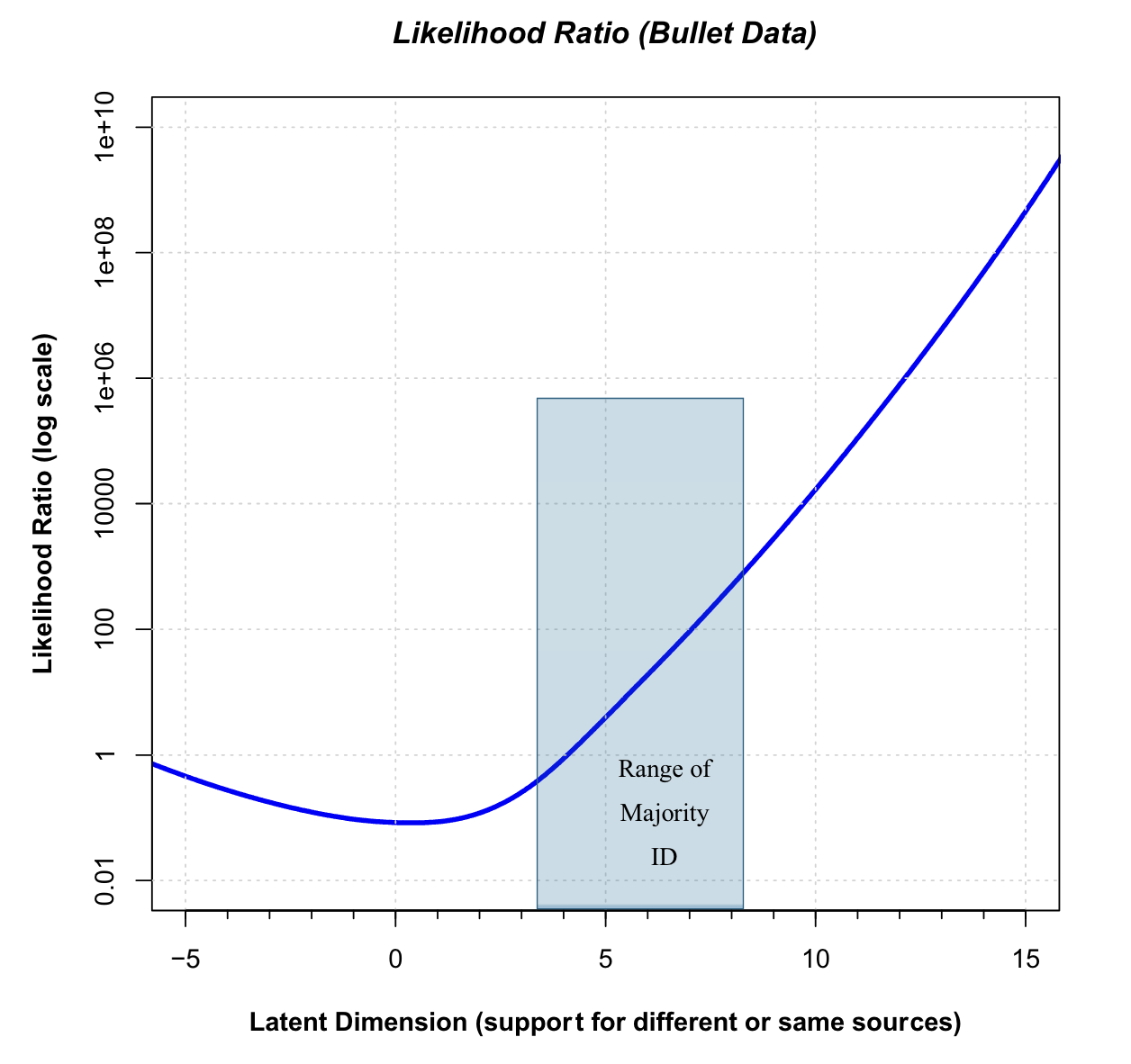

Supplement: Supplementary file 5 — Figure S5. [file JFO-70-84-s009.png]

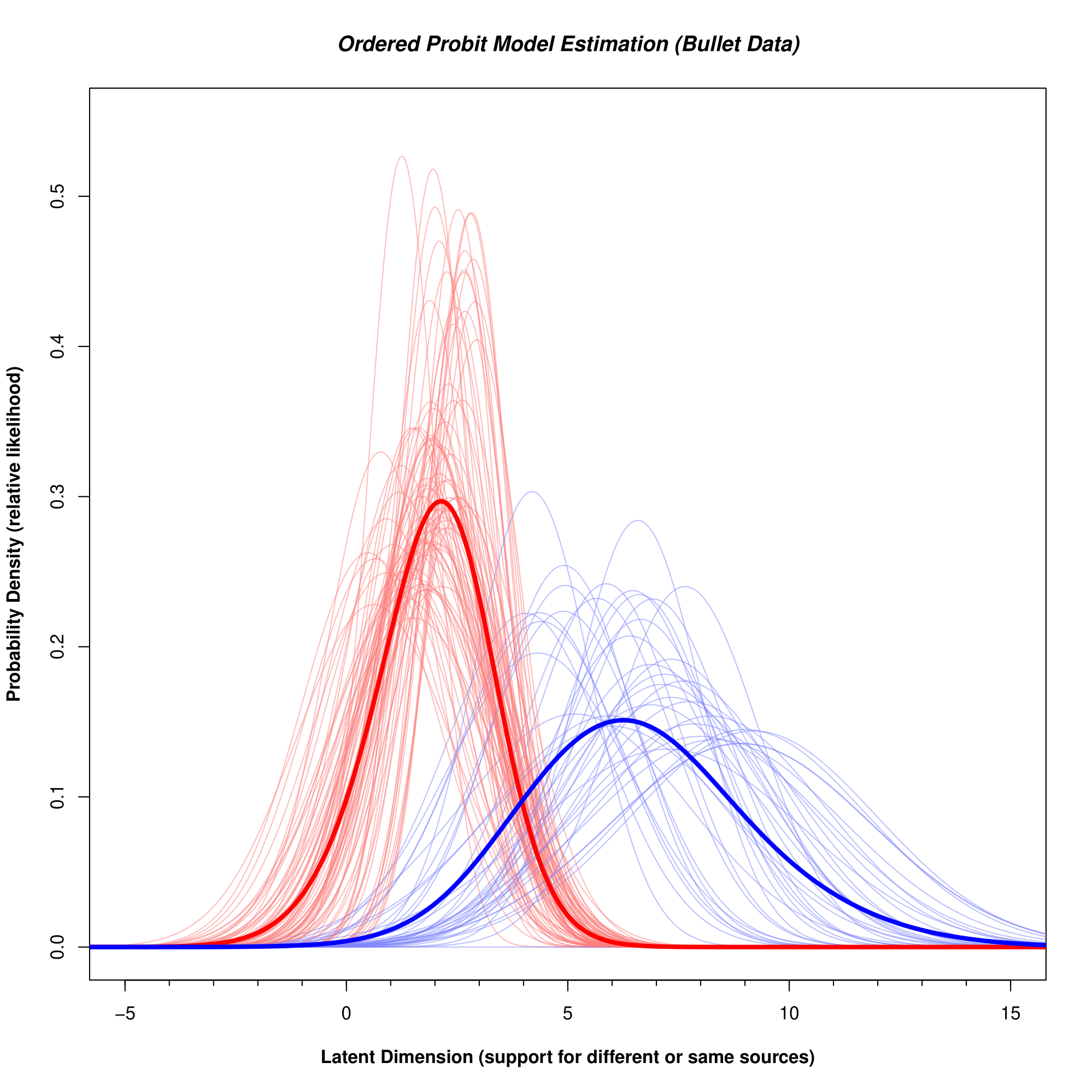

Supplement: Supplementary file 6 — Figure S6. [file JFO-70-84-s010.png]

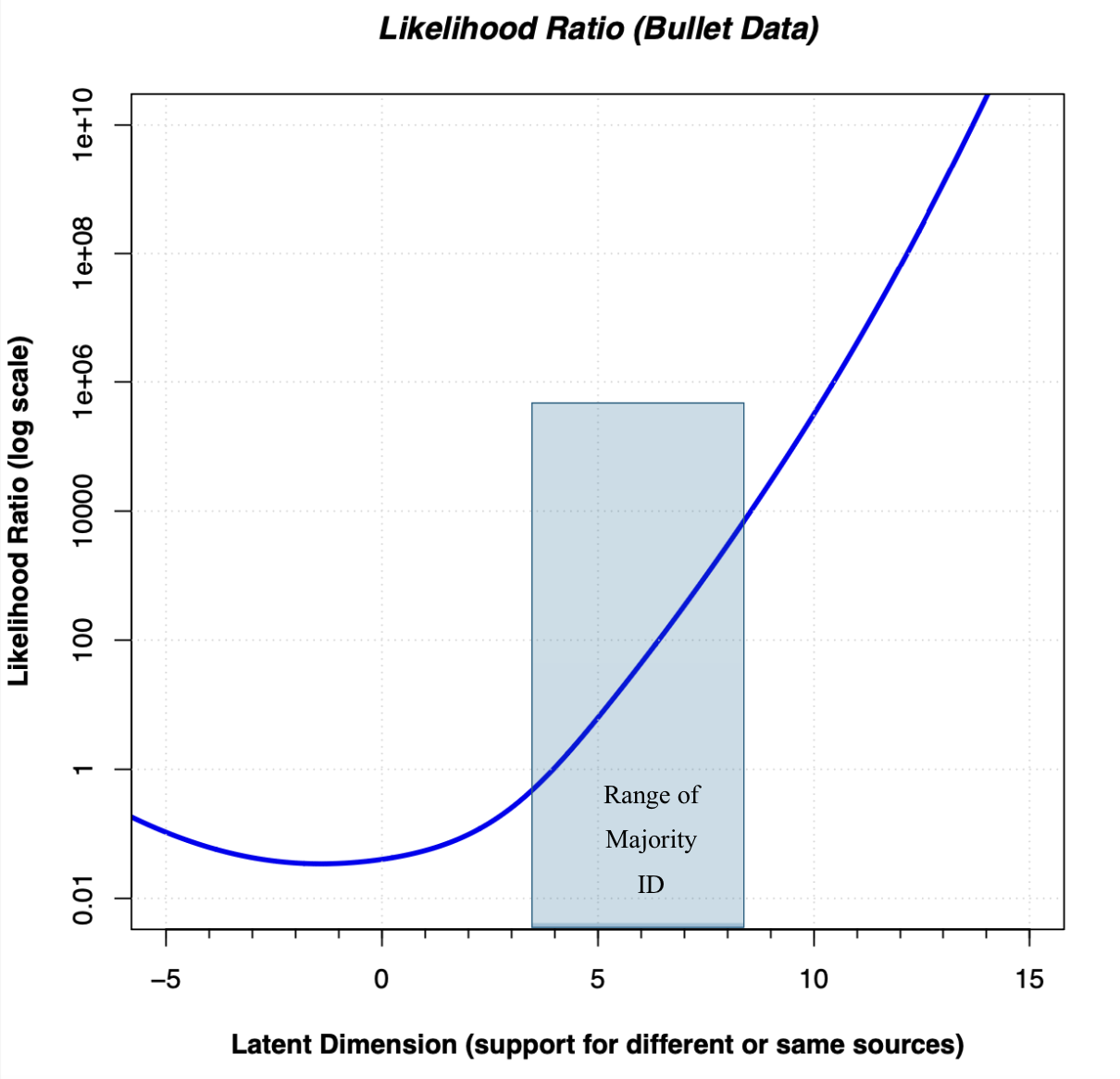

Supplement: Supplementary file 7 — Figure S7. [file JFO-70-84-s003.png]

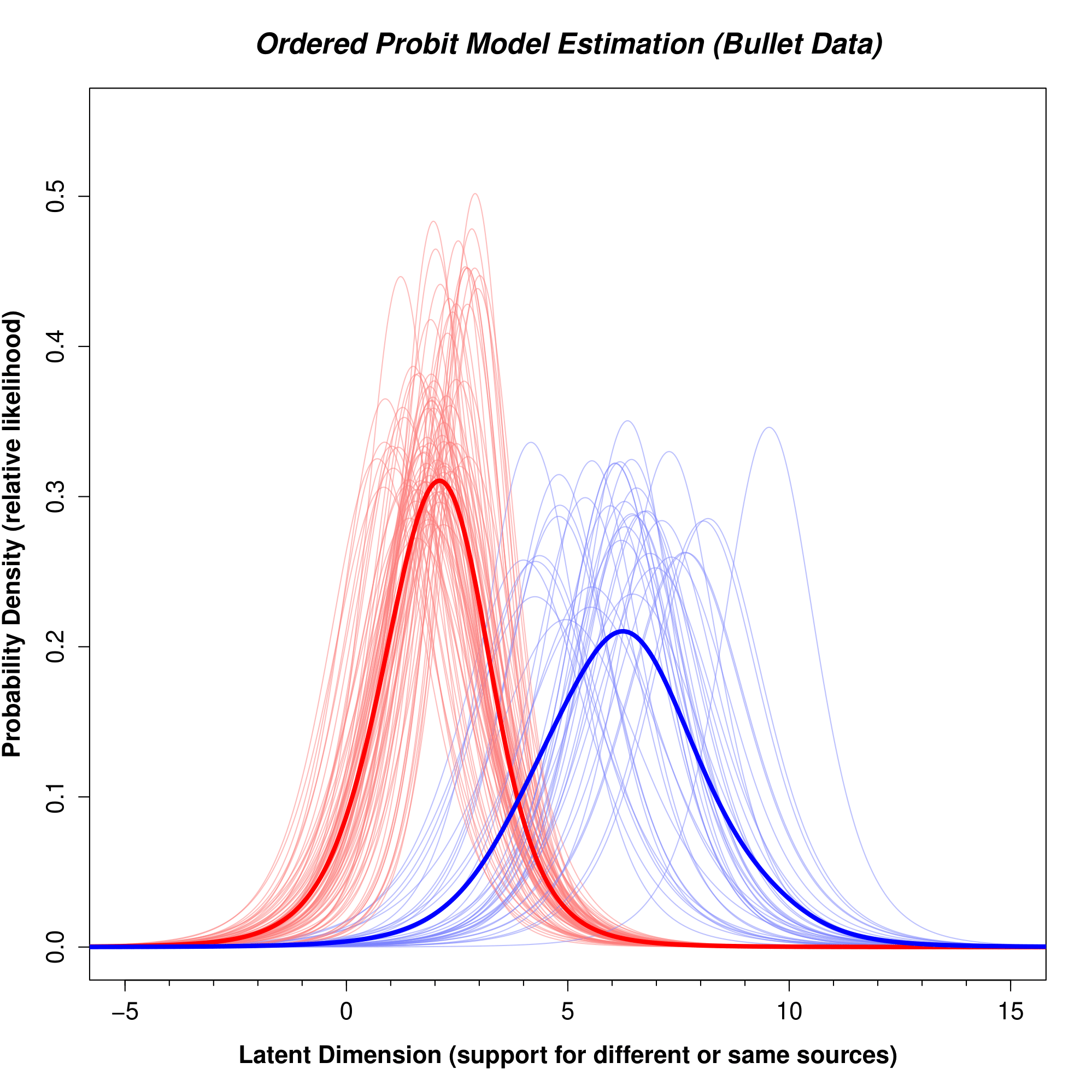

Supplement: Supplementary file 8 — Figure S8. [file JFO-70-84-s011.png]

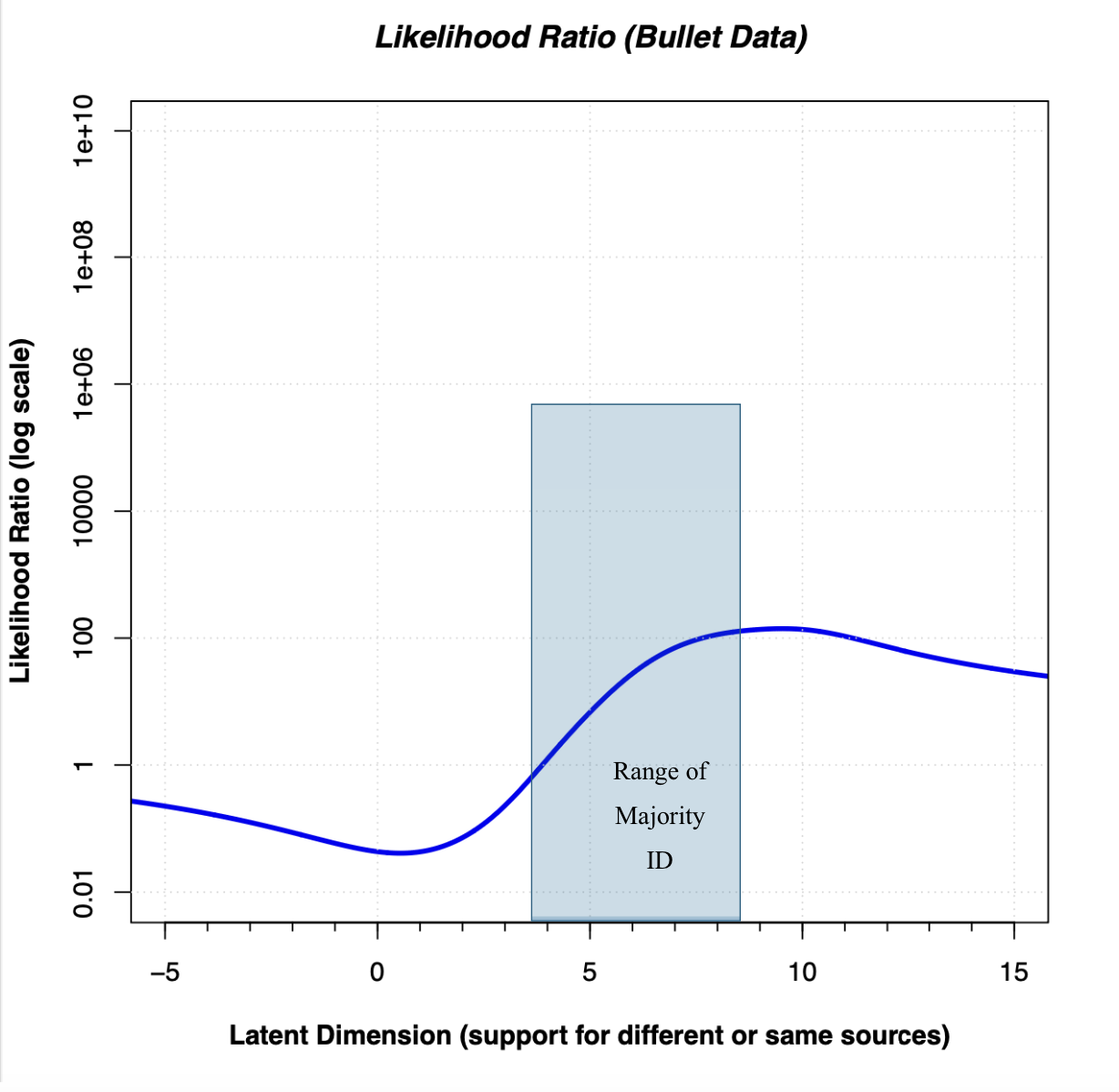

Supplement: Supplementary file 9 — Figure S9. [file JFO-70-84-s008.png]
