# Supplementary material for: Quantifying the strength of firearms comparisons based on error rate studies
Source: J Forensic Sci. 2024 Oct 30;70(1):84–97. doi: 10.1111/1556-4029.15646 (PMC11693517; doi:10.1111/1556-4029.15646)
Supplement: Supplementary file 10 — Table S1. [file JFO-70-84-s005.docx]

TABLE S1 Representative data from the Bullet data from Monson Error Rate investigation. We calculated the µ and σ value using the ordered probit model, and sorted the pairs from the lowest µ to the highest µ. The numbers on the right side of the table represent the number of examiners who responded with an Individualization (ID), Elimination (Elim), or Inconclusive (Inc-C, Inc-B, Inc-A). Each pair's ground truth is indicated by the column “Mated” with False referring to nonmated pairs and True referring to mated pairs. Bold likelihood ratio values are those pairs in which examiners gave more Identification decisions than all other responses, which reflects those comparisons that might be considered casework-like quality.

| **pairID** | **Mated** | **mu** | **sigma** | **LR** | **Elim** | **Inc-C** | **Inc-B** | **Inc-A** | **ID** | **Majority ID** |
| --- | --- | --- | --- | --- | --- | --- | --- | --- | --- | --- |
| I-Q | FALSE | 0.54 | 1.48 | 0.04 | 14 | 3 | 2 | 0 | 0 | FALSE |
| J-Q | FALSE | 0.69 | 1.68 | 0.05 | 11 | 3 | 1 | 1 | 0 | FALSE |
| L-T | FALSE | 0.70 | 1.50 | 0.05 | 13 | 5 | 0 | 1 | 0 | FALSE |
| K-S | FALSE | 0.78 | 1.21 | 0.05 | 14 | 5 | 1 | 0 | 0 | FALSE |
| V-C | FALSE | 0.93 | 1.38 | 0.05 | 13 | 4 | 3 | 0 | 0 | FALSE |
| F-K | FALSE | 0.95 | 1.56 | 0.05 | 10 | 4 | 1 | 1 | 0 | FALSE |
| 7-10 | FALSE | 1.08 | 1.46 | 0.05 | 10 | 5 | 1 | 1 | 0 | FALSE |
| W-D | FALSE | 1.20 | 1.32 | 0.06 | 11 | 7 | 1 | 1 | 0 | FALSE |
| 7-11 | FALSE | 1.22 | 0.82 | 0.06 | 11 | 8 | 0 | 0 | 0 | FALSE |
| M-U | FALSE | 1.24 | 1.24 | 0.06 | 10 | 5 | 3 | 0 | 0 | FALSE |
| J-R | FALSE | 1.34 | 1.67 | 0.06 | 11 | 3 | 4 | 2 | 0 | FALSE |
| T-A | FALSE | 1.35 | 1.56 | 0.06 | 10 | 5 | 2 | 2 | 0 | FALSE |
| Y-G | FALSE | 1.38 | 1.63 | 0.06 | 11 | 7 | 2 | 1 | 1 | FALSE |
| Z-H | FALSE | 1.48 | 1.16 | 0.07 | 10 | 9 | 2 | 1 | 0 | FALSE |
| Q-Y | FALSE | 1.52 | 1.56 | 0.07 | 11 | 4 | 5 | 2 | 0 | FALSE |
| O-W | FALSE | 1.53 | 1.50 | 0.07 | 10 | 3 | 6 | 1 | 0 | FALSE |
| H-P | FALSE | 1.55 | 1.77 | 0.07 | 9 | 4 | 1 | 4 | 0 | FALSE |
| D-M | FALSE | 1.56 | 1.16 | 0.07 | 10 | 9 | 3 | 1 | 0 | FALSE |
| N-T | FALSE | 1.61 | 1.59 | 0.07 | 8 | 1 | 6 | 1 | 0 | FALSE |
| X-E | FALSE | 1.64 | 1.16 | 0.07 | 7 | 8 | 2 | 1 | 0 | FALSE |
| P-X | FALSE | 1.64 | 1.46 | 0.08 | 10 | 5 | 5 | 2 | 0 | FALSE |
| 2-7 | FALSE | 1.70 | 1.33 | 0.08 | 8 | 5 | 5 | 1 | 0 | FALSE |
| G-O | FALSE | 1.73 | 1.62 | 0.08 | 7 | 4 | 2 | 3 | 0 | FALSE |
| X-F | FALSE | 1.74 | 1.45 | 0.08 | 9 | 8 | 4 | 1 | 1 | FALSE |
| T-B | FALSE | 1.77 | 1.39 | 0.08 | 8 | 8 | 2 | 3 | 0 | FALSE |
| W-E | FALSE | 1.78 | 1.47 | 0.08 | 9 | 6 | 4 | 3 | 0 | FALSE |
| M-V | FALSE | 1.78 | 1.30 | 0.08 | 7 | 5 | 5 | 1 | 0 | FALSE |
| S-AA | FALSE | 1.79 | 1.47 | 0.08 | 8 | 6 | 3 | 3 | 0 | FALSE |
| D-L | FALSE | 1.80 | 1.64 | 0.08 | 7 | 3 | 3 | 3 | 0 | FALSE |
| H-Q | FALSE | 1.81 | 1.41 | 0.08 | 8 | 5 | 5 | 2 | 0 | FALSE |
| E-L | FALSE | 1.81 | 1.51 | 0.08 | 6 | 5 | 4 | 0 | 1 | FALSE |
| 1-6 | FALSE | 1.83 | 1.28 | 0.09 | 10 | 5 | 9 | 1 | 0 | FALSE |
| V-D | FALSE | 1.86 | 1.31 | 0.09 | 7 | 8 | 5 | 0 | 1 | FALSE |
| K-T | FALSE | 1.87 | 1.43 | 0.09 | 8 | 6 | 4 | 3 | 0 | FALSE |
| L-U | FALSE | 1.88 | 1.65 | 0.09 | 7 | 4 | 2 | 4 | 0 | FALSE |
| A-J | FALSE | 1.89 | 0.95 | 0.09 | 6 | 8 | 6 | 0 | 0 | FALSE |
| 3-7 | FALSE | 1.91 | 1.11 | 0.09 | 7 | 8 | 6 | 1 | 0 | FALSE |
| U-B | FALSE | 1.91 | 1.18 | 0.09 | 6 | 9 | 3 | 2 | 0 | FALSE |
| C-K | FALSE | 1.91 | 1.19 | 0.09 | 5 | 6 | 4 | 1 | 0 | FALSE |
| Y-F | FALSE | 1.91 | 1.19 | 0.09 | 5 | 6 | 4 | 1 | 0 | FALSE |
| G-P | FALSE | 1.94 | 1.19 | 0.09 | 7 | 6 | 7 | 1 | 0 | FALSE |
| C-L | FALSE | 1.96 | 0.80 | 0.09 | 5 | 11 | 6 | 0 | 0 | FALSE |
| 10-3 | FALSE | 1.96 | 1.47 | 0.09 | 8 | 2 | 8 | 2 | 0 | FALSE |
| B-K | FALSE | 1.97 | 1.13 | 0.09 | 6 | 7 | 6 | 1 | 0 | FALSE |
| N-W | FALSE | 1.98 | 1.20 | 0.10 | 5 | 8 | 3 | 2 | 0 | FALSE |
| F-O | FALSE | 2.00 | 0.84 | 0.10 | 5 | 10 | 7 | 0 | 0 | FALSE |
| N-V | FALSE | 2.05 | 1.36 | 0.10 | 6 | 6 | 4 | 3 | 0 | FALSE |
| R-Z | FALSE | 2.06 | 1.49 | 0.10 | 8 | 4 | 6 | 4 | 0 | FALSE |
| J-P | FALSE | 2.08 | 1.42 | 0.10 | 5 | 5 | 3 | 3 | 0 | FALSE |
| 11-5 | FALSE | 2.08 | 1.48 | 0.10 | 10 | 4 | 11 | 2 | 1 | FALSE |
| 4-7 | FALSE | 2.10 | 0.88 | 0.11 | 3 | 7 | 6 | 0 | 0 | FALSE |
| K-Q | FALSE | 2.11 | 1.27 | 0.11 | 5 | 3 | 7 | 1 | 0 | FALSE |
| 10-4 | FALSE | 2.15 | 1.20 | 0.11 | 9 | 5 | 13 | 2 | 0 | FALSE |
| R-AA | FALSE | 2.16 | 1.39 | 0.11 | 6 | 4 | 6 | 3 | 0 | FALSE |
| U-C | FALSE | 2.18 | 1.63 | 0.11 | 7 | 5 | 4 | 4 | 1 | FALSE |
| I-R | FALSE | 2.19 | 1.36 | 0.11 | 6 | 6 | 5 | 4 | 0 | FALSE |
| P-Y | FALSE | 2.20 | 1.29 | 0.11 | 5 | 6 | 5 | 3 | 0 | FALSE |
| AA-I | FALSE | 2.24 | 1.15 | 0.12 | 5 | 6 | 8 | 2 | 0 | FALSE |
| E-N | FALSE | 2.26 | 1.40 | 0.12 | 5 | 8 | 4 | 3 | 1 | FALSE |
| 10-2 | FALSE | 2.28 | 0.91 | 0.12 | 3 | 5 | 9 | 0 | 0 | FALSE |
| J-S | FALSE | 2.29 | 1.43 | 0.12 | 6 | 5 | 5 | 5 | 0 | FALSE |
| S-A | FALSE | 2.30 | 1.37 | 0.13 | 6 | 4 | 7 | 4 | 0 | FALSE |
| O-X | FALSE | 2.31 | 1.29 | 0.13 | 4 | 5 | 5 | 3 | 0 | FALSE |
| 2-6 | FALSE | 2.31 | 1.08 | 0.13 | 4 | 8 | 10 | 0 | 1 | FALSE |
| 6-10 | FALSE | 2.34 | 1.33 | 0.13 | 5 | 4 | 9 | 1 | 1 | FALSE |
| 11-4 | FALSE | 2.34 | 1.22 | 0.13 | 5 | 6 | 10 | 1 | 1 | FALSE |
| 7-1 | FALSE | 2.43 | 0.97 | 0.14 | 3 | 10 | 9 | 3 | 0 | FALSE |
| 8-1 | FALSE | 2.43 | 1.11 | 0.14 | 4 | 4 | 10 | 2 | 0 | FALSE |
| 1-5 | FALSE | 2.46 | 0.95 | 0.15 | 3 | 7 | 11 | 2 | 0 | FALSE |
| 6-9 | FALSE | 2.52 | 1.33 | 0.15 | 3 | 5 | 6 | 2 | 1 | FALSE |
| Q-Z | FALSE | 2.53 | 1.33 | 0.16 | 4 | 6 | 4 | 6 | 0 | FALSE |
| 8-11 | FALSE | 2.53 | 0.83 | 0.16 | 1 | 6 | 9 | 1 | 0 | FALSE |
| 3-8 | FALSE | 2.62 | 1.11 | 0.17 | 3 | 5 | 9 | 4 | 0 | FALSE |
| 5-9 | FALSE | 2.66 | 0.91 | 0.18 | 2 | 2 | 12 | 1 | 0 | FALSE |
| 4-8 | FALSE | 2.66 | 0.91 | 0.18 | 2 | 2 | 12 | 1 | 0 | FALSE |
| 9-1 | FALSE | 2.69 | 0.88 | 0.18 | 1 | 5 | 10 | 2 | 0 | FALSE |
| 9-2 | FALSE | 2.69 | 0.96 | 0.18 | 2 | 3 | 11 | 2 | 0 | FALSE |
| 5-8 | FALSE | 2.72 | 1.35 | 0.19 | 3 | 3 | 7 | 3 | 1 | FALSE |
| 4-9 | FALSE | 2.81 | 0.83 | 0.21 | 1 | 3 | 12 | 2 | 0 | FALSE |
| 6-11 | FALSE | 2.83 | 0.84 | 0.22 | 2 | 1 | 16 | 2 | 0 | FALSE |
| 5-10 | FALSE | 2.87 | 0.89 | 0.22 | 1 | 3 | 11 | 3 | 0 | FALSE |
| 8-2 | FALSE | 2.91 | 0.94 | 0.24 | 2 | 4 | 14 | 6 | 0 | FALSE |
| 9-3 | FALSE | 2.95 | 1.00 | 0.25 | 3 | 0 | 18 | 3 | 1 | FALSE |
| 11-11 | TRUE | 4.04 | 1.77 | 1.20 | 3 | 4 | 14 | 6 | 19 | FALSE |
| 9-9 | TRUE | 4.19 | 1.31 | 1.55 | 1 | 1 | 14 | 11 | 18 | FALSE |
| 2-2 | TRUE | 4.31 | 1.99 | 1.91 | 3 | 7 | 9 | 7 | 24 | FALSE |
| 8-8 | TRUE | 4.35 | 1.76 | 2.04 | 2 | 3 | 18 | 4 | 26 | FALSE |
| 4-4 | TRUE | 4.38 | 1.81 | 2.17 | 3 | 4 | 9 | 11 | 24 | FALSE |
| 6-6 | TRUE | 4.88 | 1.75 | **5.48** | 2 | 2 | 6 | 12 | 30 | TRUE |
| 10-10 | TRUE | 4.89 | 1.55 | **5.59** | 1 | 1 | 7 | 11 | 29 | TRUE |
| 5-5 | TRUE | 4.92 | 1.63 | **5.92** | 1 | 1 | 10 | 9 | 32 | TRUE |
| 3-3 | TRUE | 5.09 | 2.46 | **8.18** | 6 | 0 | 7 | 8 | 32 | TRUE |
| 1-1 | TRUE | 5.60 | 1.67 | **22.28** | 0 | 1 | 6 | 6 | 39 | TRUE |
| X-X | TRUE | 5.79 | 2.52 | **32.63** | 3 | 0 | 1 | 4 | 21 | TRUE |
| H-H | TRUE | 5.80 | 2.44 | **33.16** | 2 | 2 | 5 | 2 | 30 | TRUE |
| 7-7 | TRUE | 5.80 | 1.60 | **33.51** | 0 | 1 | 3 | 7 | 42 | TRUE |
| S-S | TRUE | 6.22 | 1.79 | **78.38** | 0 | 0 | 3 | 1 | 22 | TRUE |
| L-L | TRUE | 6.28 | 1.56 | **89.65** | 0 | 0 | 1 | 2 | 21 | TRUE |
| Y-Y | TRUE | 6.41 | 1.57 | **115.88** | 0 | 0 | 1 | 2 | 24 | TRUE |
| O-O | TRUE | 6.44 | 1.33 | **124.58** | 0 | 0 | 0 | 3 | 36 | TRUE |
| B-B | TRUE | 6.46 | 1.72 | **129.70** | 0 | 1 | 0 | 4 | 35 | TRUE |
| K-K | TRUE | 6.51 | 2.24 | **144.13** | 1 | 0 | 1 | 1 | 17 | TRUE |
| U-U | TRUE | 6.61 | 1.94 | **177.20** | 0 | 0 | 4 | 0 | 29 | TRUE |
| C-C | TRUE | 6.69 | 1.60 | **209.43** | 0 | 0 | 1 | 2 | 33 | TRUE |
| AA-AA | TRUE | 6.83 | 2.08 | **283.82** | 1 | 0 | 0 | 3 | 29 | TRUE |
| E-E | TRUE | 6.83 | 2.71 | **284.13** | 2 | 1 | 0 | 1 | 23 | TRUE |
| D-D | TRUE | 6.85 | 2.00 | **291.99** | 0 | 1 | 1 | 1 | 26 | TRUE |
| M-M | TRUE | 6.95 | 2.17 | **366.19** | 1 | 0 | 0 | 2 | 24 | TRUE |
| P-P | TRUE | 7.00 | 1.89 | **402.59** | 0 | 1 | 0 | 2 | 32 | TRUE |
| Z-Z | TRUE | 7.23 | 1.48 | **661.42** | 0 | 0 | 0 | 1 | 27 | TRUE |
| A-A | TRUE | 7.23 | 2.03 | **663.46** | 0 | 1 | 1 | 1 | 36 | TRUE |
| I-I | TRUE | 7.31 | 2.18 | **787.47** | 1 | 0 | 0 | 2 | 32 | TRUE |
| W-W | TRUE | 7.34 | 2.39 | **852.73** | 1 | 0 | 2 | 0 | 30 | TRUE |
| N-N | TRUE | 7.44 | 2.53 | **1042.96** | 1 | 1 | 1 | 0 | 30 | TRUE |
| F-F | TRUE | 7.62 | 2.28 | **1549.36** | 1 | 0 | 0 | 1 | 28 | TRUE |
| Q-Q | TRUE | 7.67 | 2.55 | **1753.93** | 1 | 1 | 0 | 0 | 26 | TRUE |
| V-V | TRUE | 8.11 | 2.57 | **4658.12** | 1 | 1 | 0 | 0 | 35 | TRUE |
| R-R | TRUE | 8.12 | 2.38 | **4778.29** | 1 | 0 | 0 | 0 | 25 | TRUE |
| G-G | TRUE | 8.15 | 2.56 | **5155.97** | 1 | 1 | 0 | 0 | 36 | TRUE |
| J-J | TRUE | 8.26 | 2.38 | **6596.64** | 1 | 0 | 0 | 0 | 28 | TRUE |
| T-T | TRUE | 9.48 | 1.33 | **121593.79** | 0 | 0 | 0 | 0 | 26 | TRUE |
