# Supplementary material for: Quantifying the strength of firearms comparisons based on error rate studies
Source: J Forensic Sci. 2024 Oct 30;70(1):84–97. doi: 10.1111/1556-4029.15646 (PMC11693517; doi:10.1111/1556-4029.15646)
Supplement: Supplementary file 11 — Table S2. [file JFO-70-84-s001.docx]

TABLE S2 Representative data from the cartridge data from Monson Black Box investigation [7]. We calculated the µ and σ value using the ordered probit model, and sorted the pairs from the lowest µ to the highest µ. The numbers on the right side of the table represent the number of examiners who responded with an Individualization (ID), Elimination (Elim), or Inconclusive (Inc-C, Inc-B, Inc-A). Each pair's ground truth is indicated by the column “Mated” with False referring to nonmated pairs and True referring to mated pairs. Bold likelihood ratio values are those pairs in which examiners gave more Identification decisions than all other responses, which reflects if a comparison might be considered casework-like quality.

| **pairID** | **Mated** | **mu** | **sigma** | **LR** | **Elim** | **Inc-C** | **Inc-B** | **Inc-A** | **ID** | **Majority ID** |
| --- | --- | --- | --- | --- | --- | --- | --- | --- | --- | --- |
| 2-7 | FALSE | -1.16 | 1.47 | 0.00 | 25 | 1 | 0 | 0 | 0 | FALSE |
| 7-1 | FALSE | -1.16 | 1.47 | 0.00 | 25 | 1 | 0 | 0 | 0 | FALSE |
| 7-11 | FALSE | -1.04 | 1.47 | 0.00 | 21 | 1 | 0 | 0 | 0 | FALSE |
| 3-7 | FALSE | -0.98 | 1.47 | 0.00 | 19 | 1 | 0 | 0 | 0 | FALSE |
| F-O | FALSE | -0.07 | 1.42 | 0.01 | 17 | 3 | 0 | 0 | 0 | FALSE |
| N-W | FALSE | -0.02 | 1.42 | 0.01 | 16 | 3 | 0 | 0 | 0 | FALSE |
| H-O | FALSE | 0.06 | 1.48 | 0.01 | 15 | 2 | 1 | 0 | 0 | FALSE |
| 4-7 | FALSE | 0.06 | 1.42 | 0.01 | 14 | 3 | 0 | 0 | 0 | FALSE |
| O-X | FALSE | 0.26 | 1.50 | 0.01 | 16 | 2 | 2 | 0 | 0 | FALSE |
| 1-6 | FALSE | 0.39 | 1.39 | 0.01 | 20 | 5 | 1 | 0 | 0 | FALSE |
| N-V | FALSE | 0.45 | 1.44 | 0.01 | 12 | 3 | 1 | 0 | 0 | FALSE |
| R-Z | FALSE | 0.57 | 1.53 | 0.02 | 12 | 1 | 3 | 0 | 0 | FALSE |
| 11-5 | FALSE | 0.72 | 1.53 | 0.02 | 19 | 4 | 3 | 1 | 0 | FALSE |
| 10-3 | FALSE | 0.79 | 1.65 | 0.02 | 17 | 2 | 3 | 2 | 0 | FALSE |
| G-N | FALSE | 0.84 | 1.32 | 0.02 | 10 | 6 | 0 | 0 | 0 | FALSE |
| Z-F | FALSE | 0.87 | 1.42 | 0.02 | 11 | 4 | 2 | 0 | 0 | FALSE |
| 8-1 | FALSE | 0.90 | 1.36 | 0.02 | 14 | 6 | 2 | 0 | 0 | FALSE |
| J-S | FALSE | 0.90 | 1.50 | 0.02 | 11 | 4 | 1 | 1 | 0 | FALSE |
| K-R | FALSE | 1.03 | 1.38 | 0.03 | 10 | 5 | 2 | 0 | 0 | FALSE |
| 1-5 | FALSE | 1.06 | 1.62 | 0.03 | 13 | 2 | 3 | 2 | 0 | FALSE |
| H-Q | FALSE | 1.07 | 1.59 | 0.03 | 13 | 1 | 5 | 1 | 0 | FALSE |
| 11-4 | FALSE | 1.15 | 1.52 | 0.03 | 14 | 3 | 5 | 1 | 0 | FALSE |
| V-A | FALSE | 1.16 | 1.45 | 0.03 | 9 | 5 | 1 | 1 | 0 | FALSE |
| 9-3 | FALSE | 1.18 | 1.53 | 0.03 | 15 | 6 | 4 | 0 | 1 | FALSE |
| Q-Z | FALSE | 1.20 | 1.45 | 0.04 | 11 | 3 | 5 | 0 | 0 | FALSE |
| E-N | FALSE | 1.23 | 1.41 | 0.04 | 10 | 4 | 4 | 0 | 0 | FALSE |
| M-V | FALSE | 1.24 | 1.44 | 0.04 | 9 | 3 | 4 | 0 | 0 | FALSE |
| 2-6 | FALSE | 1.29 | 1.45 | 0.04 | 12 | 5 | 4 | 1 | 0 | FALSE |
| 8-11 | FALSE | 1.37 | 1.44 | 0.04 | 8 | 5 | 2 | 1 | 0 | FALSE |
| L-S | FALSE | 1.37 | 1.44 | 0.04 | 8 | 5 | 2 | 1 | 0 | FALSE |
| I-P | FALSE | 1.41 | 1.44 | 0.05 | 9 | 5 | 3 | 1 | 0 | FALSE |
| 9-2 | FALSE | 1.43 | 1.55 | 0.05 | 12 | 3 | 5 | 2 | 0 | FALSE |
| I-R | FALSE | 1.44 | 1.57 | 0.05 | 10 | 2 | 4 | 2 | 0 | FALSE |
| B-K | FALSE | 1.49 | 1.36 | 0.05 | 10 | 5 | 6 | 0 | 0 | FALSE |
| J-R | FALSE | 1.50 | 1.43 | 0.05 | 8 | 5 | 3 | 1 | 0 | FALSE |
| A-J | FALSE | 1.50 | 1.45 | 0.05 | 11 | 4 | 6 | 1 | 0 | FALSE |
| C-J | FALSE | 1.51 | 1.48 | 0.05 | 8 | 3 | 4 | 1 | 0 | FALSE |
| X-E | FALSE | 1.52 | 1.35 | 0.05 | 7 | 5 | 4 | 0 | 0 | FALSE |
| 10-4 | FALSE | 1.60 | 1.50 | 0.06 | 12 | 9 | 3 | 2 | 1 | FALSE |
| K-S | FALSE | 1.61 | 1.38 | 0.06 | 7 | 6 | 3 | 1 | 0 | FALSE |
| D-M | FALSE | 1.63 | 1.30 | 0.06 | 9 | 9 | 4 | 1 | 0 | FALSE |
| K-T | FALSE | 1.64 | 1.46 | 0.06 | 8 | 5 | 3 | 2 | 0 | FALSE |
| 4-9 | FALSE | 1.65 | 1.55 | 0.06 | 13 | 5 | 5 | 4 | 0 | FALSE |
| F-M | FALSE | 1.68 | 1.40 | 0.07 | 7 | 5 | 4 | 1 | 0 | FALSE |
| 3-6 | FALSE | 1.68 | 1.40 | 0.07 | 8 | 5 | 5 | 1 | 0 | FALSE |
| S-A | FALSE | 1.73 | 1.41 | 0.07 | 8 | 4 | 6 | 1 | 0 | FALSE |
| L-T | FALSE | 1.76 | 1.30 | 0.07 | 5 | 8 | 2 | 1 | 0 | FALSE |
| X-F | FALSE | 1.76 | 1.52 | 0.08 | 8 | 4 | 3 | 3 | 0 | FALSE |
| 1-4 | FALSE | 1.77 | 1.34 | 0.08 | 7 | 4 | 7 | 0 | 0 | FALSE |
| Y-E | FALSE | 1.79 | 1.46 | 0.08 | 7 | 2 | 6 | 1 | 0 | FALSE |
| V-C | FALSE | 1.79 | 1.35 | 0.08 | 6 | 6 | 4 | 1 | 0 | FALSE |
| 2-5 | FALSE | 1.83 | 1.40 | 0.08 | 7 | 4 | 6 | 1 | 0 | FALSE |
| U-C | FALSE | 1.84 | 1.37 | 0.08 | 7 | 5 | 6 | 1 | 0 | FALSE |
| 3-8 | FALSE | 1.84 | 1.51 | 0.08 | 11 | 4 | 9 | 1 | 1 | FALSE |
| L-U | FALSE | 1.84 | 1.40 | 0.08 | 6 | 4 | 5 | 1 | 0 | FALSE |
| AA-I | FALSE | 1.87 | 1.31 | 0.09 | 7 | 7 | 6 | 1 | 0 | FALSE |
| J-Q | FALSE | 1.89 | 1.45 | 0.09 | 7 | 6 | 3 | 3 | 0 | FALSE |
| T-B | FALSE | 1.91 | 1.24 | 0.09 | 5 | 9 | 4 | 1 | 0 | FALSE |
| W-D | FALSE | 1.92 | 1.39 | 0.09 | 6 | 6 | 4 | 2 | 0 | FALSE |
| G-P | FALSE | 1.98 | 1.44 | 0.10 | 8 | 3 | 8 | 2 | 0 | FALSE |
| W-C | FALSE | 1.98 | 1.58 | 0.10 | 7 | 2 | 3 | 4 | 0 | FALSE |
| 5-10 | FALSE | 1.98 | 1.53 | 0.10 | 9 | 6 | 5 | 3 | 1 | FALSE |
| T-A | FALSE | 2.04 | 1.49 | 0.11 | 6 | 3 | 4 | 3 | 0 | FALSE |
| 5-8 | FALSE | 2.04 | 1.47 | 0.11 | 6 | 4 | 4 | 3 | 0 | FALSE |
| I-Q | FALSE | 2.05 | 1.60 | 0.11 | 7 | 2 | 5 | 2 | 1 | FALSE |
| R-AA | FALSE | 2.08 | 1.36 | 0.12 | 5 | 8 | 3 | 3 | 0 | FALSE |
| Y-G | FALSE | 2.14 | 1.42 | 0.13 | 5 | 7 | 2 | 4 | 0 | FALSE |
| W-E | FALSE | 2.14 | 1.25 | 0.13 | 5 | 4 | 10 | 0 | 0 | FALSE |
| 6-10 | FALSE | 2.16 | 1.27 | 0.13 | 4 | 8 | 5 | 2 | 0 | FALSE |
| AA-G | FALSE | 2.19 | 1.36 | 0.14 | 4 | 5 | 5 | 2 | 0 | FALSE |
| 8-2 | FALSE | 2.20 | 1.37 | 0.14 | 8 | 6 | 9 | 4 | 0 | FALSE |
| U-A | FALSE | 2.21 | 1.30 | 0.14 | 3 | 7 | 4 | 2 | 0 | FALSE |
| 4-8 | FALSE | 2.23 | 1.42 | 0.14 | 5 | 7 | 5 | 2 | 1 | FALSE |
| 6-11 | FALSE | 2.27 | 1.62 | 0.15 | 9 | 5 | 8 | 2 | 3 | FALSE |
| 5-9 | FALSE | 2.28 | 1.18 | 0.15 | 4 | 7 | 10 | 1 | 0 | FALSE |
| P-Y | FALSE | 2.29 | 1.33 | 0.15 | 5 | 4 | 9 | 2 | 0 | FALSE |
| V-D | FALSE | 2.33 | 1.17 | 0.16 | 3 | 7 | 9 | 1 | 0 | FALSE |
| Q-Y | FALSE | 2.39 | 1.44 | 0.18 | 4 | 3 | 7 | 1 | 1 | FALSE |
| Z-H | FALSE | 2.49 | 1.30 | 0.20 | 3 | 4 | 10 | 0 | 1 | FALSE |
| D-K | FALSE | 2.49 | 1.26 | 0.21 | 3 | 2 | 10 | 1 | 0 | FALSE |
| P-X | FALSE | 2.51 | 1.29 | 0.21 | 2 | 5 | 8 | 0 | 1 | FALSE |
| V-B | FALSE | 2.58 | 1.33 | 0.23 | 2 | 5 | 7 | 1 | 1 | FALSE |
| C-L | FALSE | 2.62 | 1.43 | 0.24 | 6 | 2 | 8 | 7 | 0 | FALSE |
| E-E | TRUE | 3.60 | 1.34 | 1.05 | 2 | 0 | 16 | 5 | 7 | FALSE |
| 6-6 | TRUE | 4.40 | 1.50 | 3.81 | 2 | 1 | 15 | 11 | 26 | FALSE |
| 8-8 | TRUE | 4.48 | 1.47 | 4.34 | 1 | 2 | 12 | 11 | 25 | FALSE |
| Z-Z | TRUE | 4.86 | 1.57 | **8.36** | 1 | 0 | 6 | 5 | 18 | TRUE |
| 9-9 | TRUE | 4.94 | 1.56 | **9.59** | 2 | 1 | 4 | 14 | 31 | TRUE |
| 5-5 | TRUE | 4.97 | 1.61 | **10.05** | 1 | 2 | 7 | 8 | 30 | TRUE |
| 3-3 | TRUE | 5.04 | 1.78 | **11.39** | 2 | 3 | 6 | 7 | 33 | TRUE |
| 11-11 | TRUE | 5.13 | 1.53 | **13.36** | 1 | 2 | 2 | 13 | 33 | TRUE |
| H-H | TRUE | 5.13 | 1.50 | **13.38** | 0 | 0 | 7 | 3 | 21 | TRUE |
| M-M | TRUE | 5.18 | 1.36 | **14.53** | 0 | 0 | 3 | 7 | 20 | TRUE |
| 4-4 | TRUE | 5.23 | 1.53 | **15.86** | 0 | 1 | 8 | 6 | 34 | TRUE |
| D-D | TRUE | 5.24 | 1.48 | **16.11** | 0 | 0 | 6 | 4 | 23 | TRUE |
| 10-10 | TRUE | 5.41 | 1.60 | **21.85** | 1 | 1 | 5 | 9 | 41 | TRUE |
| I-I | TRUE | 5.41 | 1.69 | **21.98** | 1 | 0 | 4 | 2 | 20 | TRUE |
| 2-2 | TRUE | 5.43 | 1.82 | **22.68** | 2 | 2 | 3 | 6 | 34 | TRUE |
| W-W | TRUE | 5.52 | 1.50 | **26.57** | 0 | 0 | 4 | 3 | 22 | TRUE |
| J-J | TRUE | 5.55 | 1.60 | **28.15** | 0 | 1 | 3 | 2 | 20 | TRUE |
| 7-7 | TRUE | 5.57 | 2.00 | **29.39** | 3 | 2 | 4 | 3 | 37 | TRUE |
| 1-1 | TRUE | 5.63 | 1.82 | **32.49** | 2 | 2 | 2 | 7 | 40 | TRUE |
| K-K | TRUE | 5.70 | 1.67 | **36.94** | 1 | 0 | 2 | 3 | 22 | TRUE |
| G-G | TRUE | 5.72 | 1.78 | **37.97** | 1 | 1 | 4 | 2 | 30 | TRUE |
| L-L | TRUE | 5.80 | 1.57 | **43.90** | 0 | 0 | 4 | 1 | 22 | TRUE |
| R-R | TRUE | 5.81 | 1.40 | **45.23** | 0 | 0 | 1 | 5 | 26 | TRUE |
| AA-AA | TRUE | 5.83 | 1.34 | **46.49** | 0 | 0 | 0 | 6 | 27 | TRUE |
| X-X | TRUE | 5.91 | 1.58 | **54.08** | 0 | 0 | 4 | 1 | 25 | TRUE |
| Q-Q | TRUE | 5.93 | 1.43 | **56.51** | 0 | 0 | 1 | 4 | 25 | TRUE |
| U-U | TRUE | 5.99 | 1.43 | **62.86** | 0 | 0 | 1 | 4 | 27 | TRUE |
| P-P | TRUE | 5.99 | 1.43 | **63.19** | 0 | 0 | 1 | 4 | 27 | TRUE |
| V-V | TRUE | 6.02 | 1.43 | **66.59** | 0 | 0 | 1 | 4 | 28 | TRUE |
| C-C | TRUE | 6.06 | 1.56 | **71.13** | 0 | 1 | 1 | 4 | 33 | TRUE |
| B-B | TRUE | 6.13 | 1.44 | **81.05** | 0 | 0 | 1 | 4 | 32 | TRUE |
| Y-Y | TRUE | 6.38 | 1.49 | **128.59** | 0 | 0 | 1 | 2 | 26 | TRUE |
| S-S | TRUE | 6.55 | 1.71 | **179.34** | 1 | 0 | 0 | 2 | 28 | TRUE |
| F-F | TRUE | 6.91 | 1.45 | **349.97** | 0 | 0 | 0 | 2 | 36 | TRUE |
| N-N | TRUE | 6.94 | 1.74 | **370.03** | 1 | 0 | 0 | 1 | 31 | TRUE |
| T-T | TRUE | 7.25 | 1.55 | **678.57** | 0 | 0 | 1 | 0 | 30 | TRUE |
| O-O | TRUE | 7.34 | 1.47 | **803.37** | 0 | 0 | 0 | 1 | 33 | TRUE |
| A-A | TRUE | 7.34 | 1.47 | **809.13** | 0 | 0 | 0 | 1 | 33 | TRUE |
