# Supplementary material for: Quantifying the strength of firearms comparisons based on error rate studies
Source: J Forensic Sci. 2024 Oct 30;70(1):84–97. doi: 10.1111/1556-4029.15646 (PMC11693517; doi:10.1111/1556-4029.15646)
Supplement: Supplementary file 12 — Data S1. [file JFO-70-84-s012.docx]

**SUPPLEMENTAL MATERIAL**

The supplemental material is organized to provide additional information on the robustness of the Ordered Probit Model. We conducted various sensitivity analyses and examined the impact of variations in the model assumptions: turning off shrinkage, increasing mu's standard deviation, and using a t-distribution instead of a normal distribution. Additionally, the material presents the response distribution for sixty random bullet and cartridge pairs. Instructions to reproduce the graphs from both the supplemental material and the main paper are available on the OSF site.

**Sensitivity Analysis**

In order to understand the robustness of the Ordered Probit Model, we conducted various sensitivity analyses and examined the impact of variations in the model assumptions.

The first sensitivity analysis we looked at is the effect of turning off shrinkage. Making the Ordered Probit Model free to fit the data without any constraint of the standard deviation lead to wider standard deviation in some cases (Figure S4). This results in complex changes in likelihood rations, increasing some at the expense of decreasing others. As a result likelihood ratios for latent values around 5 to 6 tend to decrease (Figure S5)

We also changed the priors by increasing μ’s standard deviation to 10*nYlevels where nYlevels is the number of conclusions (5). As a result, we observed that the μ values shifted to the right in pairs with LRs around 400 and that it resulted in a wider distribution for the means. This increased in the standard deviation of the priors on μ allowed the model to be less constrained by prior beliefs when estimating the parameters (Figure S6). This led to the likelihood ratio curve showing a marginal increase, reflecting the increased uncertainty introduced by the wider priors. Likelihood ratios on the edge drop while likelihood ratios coming from unanimous bullet comparisons have their likelihood ratios increase (Figure S7).

Finally, we ran the model using a t-distribution instead of a normal distribution. The t-distribution has heavier tails compared to the normal distribution. This means that extreme values are more likely under a t-distribution, leading to a higher probability of observing outliers or extreme events in the data (Figure S8). When it comes to likelihood ratio values, they are influenced by the heavy tails, leading to a peak value before the probabilities start to decrease as you move further away from the mean (Figure S9).

The sensitivity analyses were done using the Bullet Data, and similar results were found on the cartridge data.

None of these changes to the assumptions underlying the ordered probit model systematically increased the likelihood ratios across all values of μ. Indeed, many of these changes increased the likelihood ratios for comparisons with larger values of μ at the expense of likelihood ratios for smaller values of μ, which is arguably worse than the original model because small likelihood ratios are of most concern when it comes to evidence interpretation. Overall these sensitivity analyses demonstrate that the modest likelihood ratios reported in the main paper are not a result of particular assumptions or choices of parameter values for prior distributions.
